# Hydrogel technologies in andrology: advances in research and prospective applications

**DOI:** 10.3389/fphar.2025.1635007

**Published:** 2025-07-28

**Authors:** Xinlei Guo, Jingyi Zhang, Yanxin Guan, Xingzhao Tian, Yanan Gong, Yao Xiao, Fang Yang, Degui Chang, Xujun Yu

**Affiliations:** ^1^TCM Regulating Metabolic Diseases Key Laboratory of Sichuan Province, Hospital of Chengdu University of Traditional Chinese Medicine, Chengdu, China; ^2^School of Medical and Life Sciences, Chengdu University of Traditional Chinese Medicine, Chengdu, China

**Keywords:** hydrogel, andrological diseases, drug delivery, tissue engineering, tumor treatment, reproductive medicine

## Abstract

Hydrogels, owing to their outstanding physicochemical properties and excellent biocompatibility, have emerged as a focal point of research and clinical application in andrological diseases. This review systematically summarizes cutting-edge progress in hydrogel applications for Prostate Cancer (PC), Bladder Cancer (BC), Erectile Dysfunction (ED), Male Reproductive Medicine (MRM), and urinary tract tissue engineering. Current studies indicate that hydrogels can serve as protective spacers in PC radiotherapy to mitigate radiation-induced rectal toxicity and as precise drug-delivery vehicles to enhance antitumor efficacy. Moreover, hydrogels demonstrate unique and broad potential in neurovascular repair, immunomodulation, sperm selection, *in vitro* spermatogenesis modeling, and tissue regeneration. Future advancements in hydrogel technology—through intelligent responsive design, integration of bioactive molecules, incorporation of advanced manufacturing processes, and rigorous translational research—are expected to significantly elevate treatment standards for andrological diseases and improve patient quality of life.

## Graphical Abstract

**Figure:**
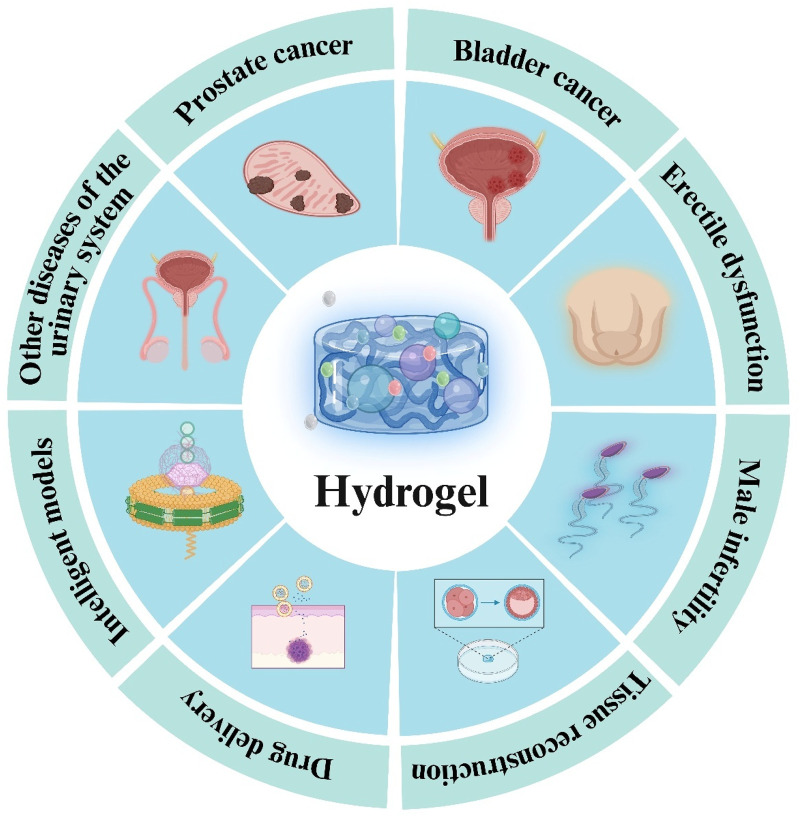
Applications of hydrogel in andrology.

## 1 Introduction

As global populations age, the incidence and mortality of andrological diseases have continued to increase (Schafer et al., 2025). Although conventional therapies have achieved substantial clinical success, significant challenges persist in managing andrological malignancies, male infertility, Erectile Dysfunction (ED), and urethral reconstruction. For example, in 2022, approximately 1,460,000 new Prostate Cancer (PC) cases were diagnosed worldwide, resulting in over 390,000 deaths; in 2020, Bladder Cancer (BC) accounted for more than 500,000 new cases—both demonstrating year-on-year increases, According to projections, the prevalence of PC is expected to reach roughly 2.4 million confirmed cases and about 712,000 deaths by 2040 (Sung et al., 2021). The steep rise in patient numbers will not only profoundly diminish patients’ quality of life but also impose immense pressure on healthcare systems worldwide (Bray et al., 2024). Despite the widespread adoption of existing treatment paradigms, the non-targeted nature of current drug-delivery systems often leads to suboptimal therapeutic outcomes and increased patient discomfort; precise control over drug release remains a critical unmet need (Londhe et al., 2025; Choi et al., 2024). In urethral tissue engineering, reconstruction is particularly hindered by mechanical deformation stresses, the harsh Urinary Microenvironment (UME), and the absence of a Regenerative Microenvironment (RME), factors that collectively inhibit repair and may result in irreversible urethral scarring (Jin et al., 2025).

Hydrogels, three-dimensional (3D) networks of crosslinked hydrophilic polymers characterized by high water content and excellent biocompatibility, mimic the natural Extracellular Matrix (ECM) (Liang Y. et al., 2022). Due to their ECM-mimetic architecture, hydrogels replicate the hydrated state of biological tissues *in vivo*, providing an ideal platform for drug delivery, regenerative medicine, and tissue repair, thereby offering new perspectives and potential solutions to the challenges faced by traditional therapies in andrology (Ho et al., 2022; Yang Y. et al., 2022; Pablos et al., 2024). In andrological oncology, hydrogels have been employed as drug-delivery systems to achieve precise locoregional release of chemotherapeutic agents, thereby elevating intratumoral drug concentrations, reducing systemic exposure, minimizing the risk of drug resistance and off-target toxicity, and enhancing antitumor efficacy (Jiang et al., 2024). For male infertility, hydrogels have been utilized as biomaterial carriers for cryopreservation of testicular tissue or cells, preserving viability with minimal damage, and supporting *in vitro* spermatogenesis modeling, which offers a potential route to fertility restoration (Tan et al., 2024). In urethral tissue engineering, hydrogel-based scaffolds have been shown to stabilize the defect site, provide a supportive cellular environment, and reconstitute the RME (Jin et al., 2025; Fischer-Valuck et al., 2017). Moreover, continued advances in nanotechnology and bioprinting are expected to enable the integration of hydrogels with intelligent and customizable therapeutic platforms (Zhao et al., 2023).

Owing to their unique physicochemical properties and excellent biocompatibility, hydrogels have demonstrated significant potential for the treatment of andrological diseases. As fabrication techniques and biomaterials science advance, hydrogels are anticipated to facilitate innovative therapeutic solutions for andrological disorders. This review systematically summarizes research progress in hydrogel applications in andrology, focusing on their roles in andrological oncology, reproductive health, and tissue engineering, and forecasts future development trends to provide a theoretical framework for related investigations (Mittal et al., 2024a).

## 2 Advantages of hydrogel characteristics in modern biomedicine

Hydrogels are formed by hydrophilic polymers through specific chemical or physical crosslinking, which enables them to absorb large volumes of water while maintaining the integrity of their 3D network structure, thereby providing a stable, continuous matrix (Tang et al., 2025). To date, hydrogels have been widely applied in biomedical engineering fields such as drug delivery, antimicrobial agents, biosensors, tissue engineering, and wound healing (Pablos et al., 2024; Li J. et al., 2024; Karmakar et al., 2024). Their principal properties include high hydration and hydrophilicity, distinctive microporous architecture, excellent biocompatibility, and tunable physicochemical characteristics (Mittal et al., 2024b).

### 2.1 High hydration, hydrophilicity, and unique microporous architecture

**FIGURE 1:**
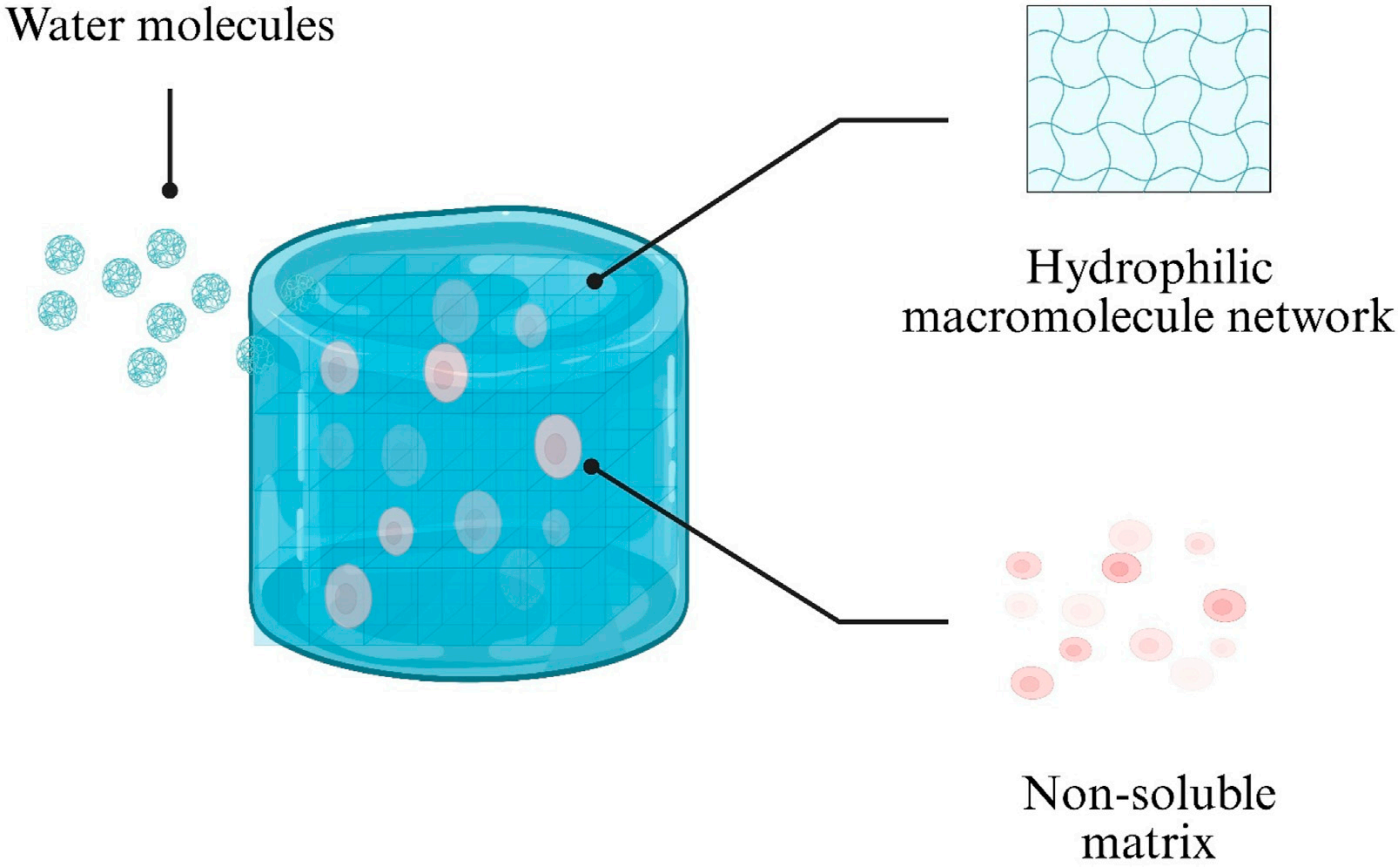
Schematic illustration of water uptake by a hydrogel. Water molecules diffuse into the hydrophilic polymer network (blue), while the non-soluble matrix (pink) maintains structural integrity.

Hydrogels are distinguished by their high hydration capacity and hydrophilicity (Figure 1). These properties arise from hydrophilic macromolecular networks formed by water-soluble polymers via chemical or physical crosslinking (Patel and Mequanint, 2011). Such networks can absorb up to 1,000-fold their dry weight in water, swelling in aqueous environments without dissolving (Chai et al., 2017). This creates an internal aqueous microenvironment that protects encapsulated cells and sensitive drug molecules, making hydrogels ideal for cell culture and drug encapsulation (Saludas et al., 2017).

The unique microporous architecture of hydrogels confers high permeability. Pore size and distribution can be precisely controlled through specific fabrication methods to achieve programmable release of loaded agents (Fazli et al., 2024). For example, incorporation of zinc oxide nanoparticles increases hydrogel porosity, water uptake capacity, thermal stability, and mechanical strength, thereby modulating the release kinetics of nettle extract and providing sustained therapeutic effects (Fazli et al., 2024). Consequently, hydrogel systems with these characteristics have been extensively utilized for drug delivery applications in andrology (Pishavar et al., 2021).

### 2.2 Biocompatibility

**FIGURE 2:**
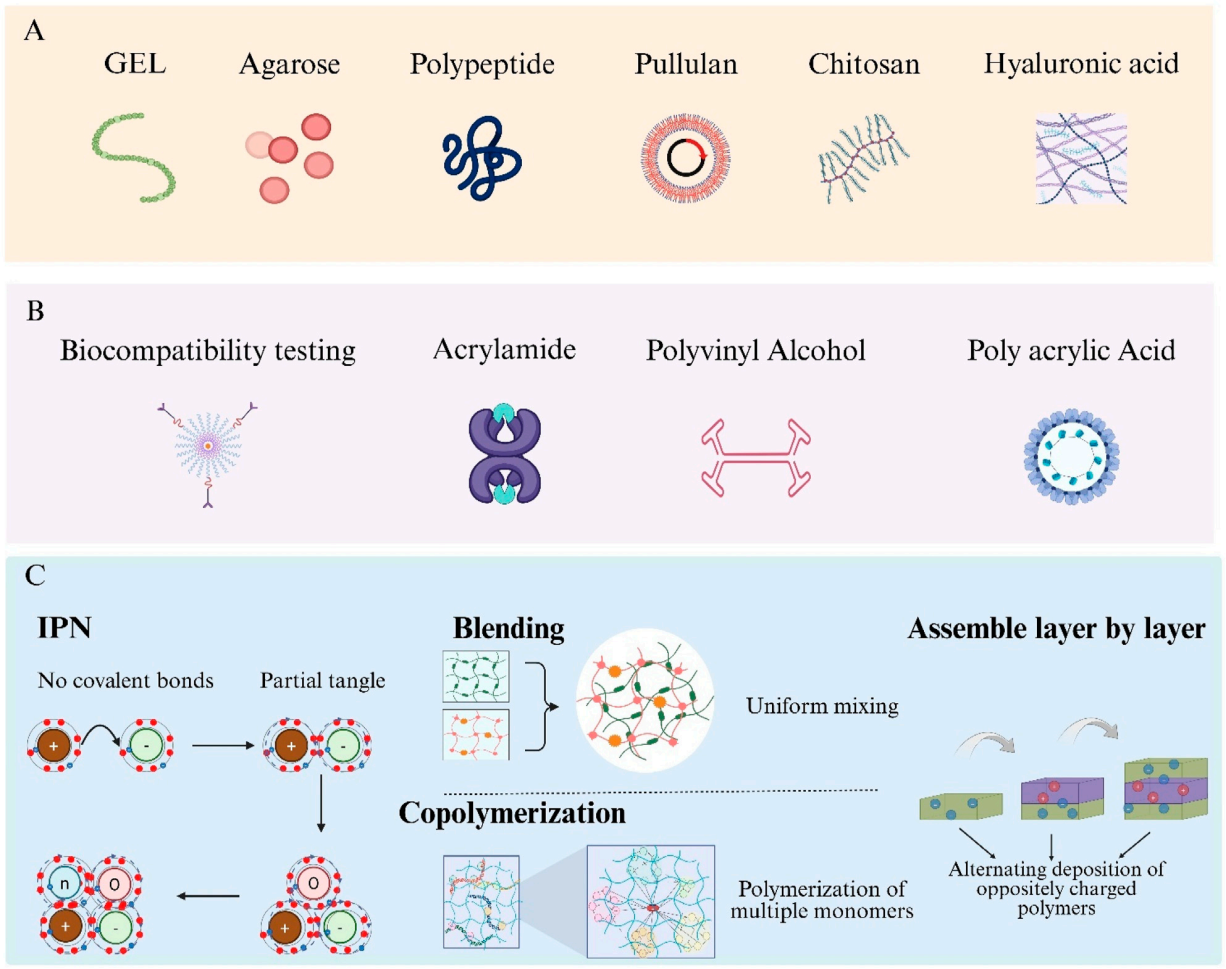
Hydrogel categories and hybrid-fabrication strategies. **(A)** Natural hydrogel formers: gelatin, agarose, polypeptide, pullulan, chitosan and hyaluronic acid. **(B)** Typical synthetic precursors: acrylamide, Polyvinyl Alcohol (PVA) and Polyacrylic Acid (PAA). **(C)** Four routes to build hybrid hydrogels—Interpenetrating Polymer Networks (IPNs), polymer blending, copolymerisation and layer-by-layer assembly.

Biocompatibility of hydrogels is primarily determined by their constituent materials. Based on material origin, hydrogels are classified as synthetic, natural, or hybrid. Synthetic hydrogels offer precise tunability and robust mechanical properties; examples include acrylamide and its derivatives, Polyvinyl Alcohol (PVA), Polyacrylic Acid (PAA), and polyurethane-based hydrogels (Figure 2A) (Sennakesavan et al., 2020; Xia and Chen, 2022; Mohammad et al., 2024; Luthfianti et al., 2024; Elliott et al., 2004). Natural hydrogels derive from biomolecules and include Gelatin (GEL), agarose, collagen, polypeptides, pullulan, Chitosan (CS), and Hyaluronic Acid (HA) (Figure 2B) (Huang et al., 2020; Su et al., 2021; Yoo et al., 2020; Wollenberg et al., 2018; Pan et al., 2021; Xu et al., 2019; Wang et al., 2022). Hybrid hydrogels integrate two or more polymer types, combining the mechanical strength of synthetic materials with the intrinsic biocompatibility of natural materials to overcome limitations such as insufficient mechanical integrity of natural hydrogels and lack of bioactivity in purely synthetic systems (Xia and Chen, 2022; Saroia et al., 2018).

Formation of hybrid hydrogels is achieved via four primary strategies: Interpenetrating Polymer Networks (IPNs), blending, copolymerization, and layer-by-layer assembly (Figure 2C) (Vasile et al., 2020; Cai et al., 2021; Shymborska et al., 2023; Park et al., 2018). IPNs consist of two or more polymer networks interlaced at the molecular scale without covalent bonds, enhancing mechanical strength and stimuli responsiveness. Blending involves uniform mixing of distinct hydrogel polymers to balance their individual advantages. Copolymerization employs multiple monomers in a single polymerization reaction, allowing precise control over network composition at the molecular level. Layer-by-layer assembly is performed by alternately depositing oppositely charged polymers to build multilayered composite structures.

Because many hydrogel materials are composed of ECM components, they inherently exhibit high biocompatibility. Moreover, by mimicking the native tissue microenvironment during fabrication, both compatibility and structural stability are further enhanced, facilitating applications in tissue engineering and regenerative medicine (Naahidi et al., 2017). In andrology, this biocompatibility has been widely harnessed for drug-delivery systems and tissue-repair scaffolds (Ren Y. et al., 2023; Moon et al., 2023).

### 2.3 Tunable physicochemical properties

**FIGURE 3:**
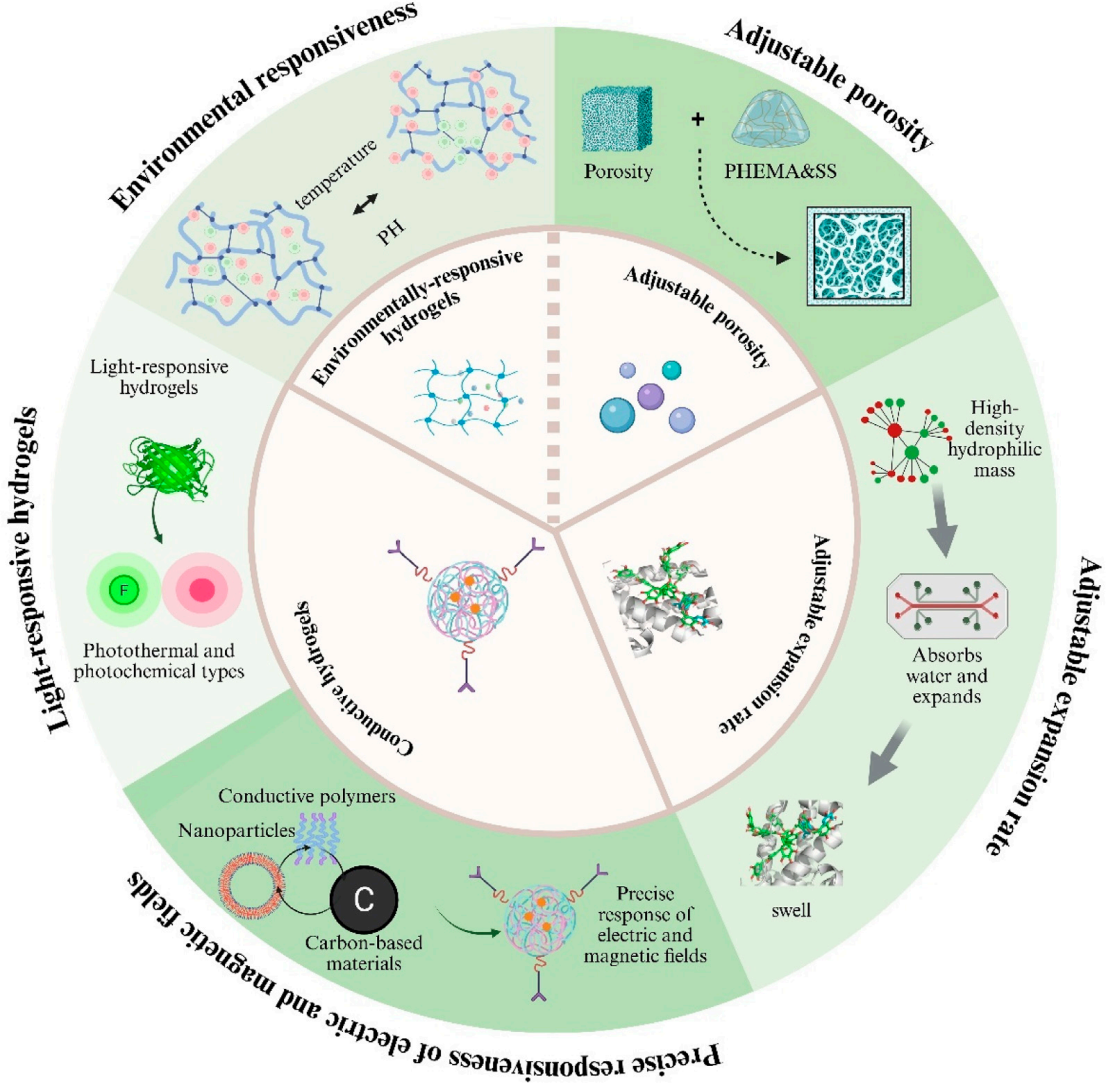
Key tunable properties of hydrogels for biomedical use. Illustrated are five adjustable features: (i) environmental responsiveness to pH or temperature; (ii) controllable porosity; (iii) variable swelling (expansion) rates; (iv) light-responsive behaviour (photothermal or photochemical); and (v) electrical/magnetic responsiveness achieved with conductive fillers.

Hydrogels, as highly tunable smart materials, have physicochemical properties that can be precisely adjusted through variations in polymer composition, network architecture, and fabrication techniques, allowing customization for diverse biomedical applications (Wang Y. et al., 2025). Controllable parameters include swelling ratio, porosity, pH-responsiveness, temperature-responsiveness, photoresponsiveness, and electrical and magnetic properties, which confer distinct advantages over conventional materials as Figure 3 (Wu et al., 2024; Maiti et al., 2024; Amirthalingam et al., 2023). These physicochemical traits not only govern the hydrogel’s physical and chemical behavior but also directly influence its functional performance in drug delivery and tissue engineering contexts (De Nitto et al., 2024; Luo et al., 2023).

#### 2.3.1 Swelling ratio

The swelling behaviour of hydrogels is jointly governed by surface pore size, network architecture and overall hydrophilicity/hydrophobicity. A high density of hydrophilic functional groups together with a macroporous structure markedly boosts swelling capacity and drug-loading efficiency, better satisfying the demands of drug delivery in andrology (Feng and Wang, 2023). However, pronounced swelling is often accompanied by macroscopic deformation and diminished mechanical strength. To overcome this, Zhou et al. designed highly cross-linked, hydrophobic hydrogels that exhibit controllable swelling in aqueous environments, showing excellent potential as bio-adhesive sealants and antibacterial agents for suture-free wound-closure systems (Zhou et al., 2019).

#### 2.3.2 Porosity

Hydrogels offer finely tunable porosity: their characteristic porous networks emulate native tissue and, under dynamic loading, deliver high mass-transport efficiency alongside excellent mechanical performance (Foudazi et al., 2023). Such architecture also accelerates *in-vivo* cell adhesion and proliferation, giving hydrogels a natural advantage in andrological applications—for example, corpus cavernosum repair for Erectile Dysfunction (ED) (Martin et al., 2021). However, excessive porosity dramatically lowers the material’s elastic modulus, resulting in greater deformation and a noticeable loss of load-bearing capacity (Argun et al., 2014). To mitigate this, researchers have shown that increasing the silk-fibroin content within a poly(2-hydroxyethyl methacrylate) matrix effectively reduces pore size, thereby optimising the hydrogel’s overall properties (Tuancharoensri et al., 2023).

#### 2.3.3 Stimuli-responsive properties and functional applications

Hydrogels can be chemically modified or supplemented with additives to endow them with reversible responses to external stimuli (Koetting et al., 2015; Liao et al., 2025). pH-responsive hydrogels, which contain acidic or basic groups, ionise and swell as environmental pH changes and are widely used for targeted drug release (Xiong et al., 2024). Thermoresponsive hydrogels take advantage of the fact that tumour sites are warmer than normal tissue, finding applications in cancer therapy and wound healing (Bordat et al., 2019). Photo-responsive hydrogels—classified as photothermal or photochemical—offer high precision, non-invasiveness and reversibility, and are primarily used in disease modelling and smart wearable devices (Homma et al., 2021).

Conductive hydrogels achieve precise responsiveness to electric and magnetic fields by incorporating conductive fillers such as metal nanoparticles, conductive polymers or carbon-based materials (Lo et al., 2021). These composites couple the intrinsic softness of hydrogels with the electron-transport capability of conductive materials. Although their conductivity is lower than that of conventional metal conductors, their unique “aqueous soft-material” architecture imparts excellent biocompatibility and tunable mechanics, giving them distinctive advantages and broad prospects in cutting-edge areas such as smart drug-delivery systems (Chen et al., 2023).

### 2.4 Performance differences among hydrogel materials

Natural hydrogels, distinguished by their unique architecture and high water content, are widely employed in andrological disease management (drug delivery) and male infertility therapy (tissue preservation, regeneration and repair) because of their excellent controlled-release performance, biocompatibility and tissue non-toxicity (Tan et al., 2024; Ahmed, 2015). Their clinical utility, however, is often constrained by weak mechanical strength and limited diffusion rates (Calvert, 2009). Synthetic hydrogels, produced through tailored fabrication techniques, can offset these shortcomings. Studies have demonstrated that double-network hydrogels—with two interpenetrating, highly cross-linked networks—exhibit orders-of-magnitude increases in strength, stiffness and toughness, thereby markedly improving the mechanical limitations of natural hydrogels (Zhang and Khademhosseini, 2017). In addition, topological design strategies that interlock polymer chains with figure-eight cross-linkers yield highly elastic hydrogels; the system developed by Wenz et al. achieved notably higher swelling capacity and fracture strain than conventional types, with a swelling ratio of up to forty-fold (Kali et al., 2016). Such high-strength synthetic hydrogels show great promise for repairing tissues exposed to harsh physiological environments, such as the urethra and bladder (Xiao et al., 2021). Nevertheless, despite advances in mechanical tuning, synthetic hydrogels often exhibit poor fatigue resistance, as irreversible breakage of intra- or intermolecular covalent bonds can occur within the network (Zhang and Khademhosseini, 2017). Given the respective limitations of natural and synthetic hydrogels, hybrid hydrogels—created by integrating multiple materials through sophisticated fabrication approaches—have emerged to provide better drug-release profiles and bioavailability (Omidian et al., 2025). This strategy introduces design complexity: different material combinations influence interfacial affinity, solubility, stability and release kinetics, thereby modulating drug-release behaviour. If processing conditions are not carefully controlled, the sustained-release potential may be diminished (Table 1) (Cirri et al., 2022).

**TABLE 1:** Advantages and disadvantages of hydrogel materials and their preferential applications in andrological diseases.

Category	Advantages	Shortcomings	Preferred andrological applications
Natural hydrogels	Excellent biocompatibility and drug-delivery capability	Low mechanical strength	Drug delivery male infertility (Tan et al., 2024; Ahmed, 2015)
Synthetic hydrogels	High mechanical strength	Poor fatigue resistance	Bladder and urethral reconstruction (Xiao et al., 2021)
Hybrid hydrogels	Improved drug release and bioavailability	Difficult-to-control release behaviour and complex fabrication	Drug delivery (Tao et al., 2019) male infertility (Rahbar et al., 2024) ED (Wang et al., 2024) andrological-disease diagnostics (Kim et al., 2021)

## 3 Therapeutic applications of hydrogels in andrological diseases

### 3.1 Applications of hydrogels in prostate cancer

PC treatment strategies aim for curative outcomes, a key challenge is the reduction of therapy-related toxicities. Owing to their outstanding biocompatibility, injectability for *in-situ* formation and tunable physicochemical responsiveness, hydrogels show unique promise in PC radiotherapy for lowering rectal toxicity, minimising side effects and enabling more precise irradiation.

#### 3.1.1 Hydrogel spacers in prostate radiotherapy

Radical prostatectomy remains the primary treatment modality for PC, yet postoperative microcirculatory changes and alterations in the immune microenvironment due to residual tumor tissue can lead to local recurrence and metastasis (Fan et al., 2022). Adjuvant External-Beam Radiotherapy (EBRT), Stereotactic Body Radiotherapy (SBRT), and focal laser ablation are employed to prevent recurrence (Zhong et al., 2021; Fujihara and Ukimura, 2022). Clinical trials have demonstrated that dose-escalated EBRT prolongs survival in PC patients (Klein and Silverman, 2008). However, because the prostate lies immediately anterior to the rectum, radiation-induced rectal toxicity is a common adverse effect (Karsh et al., 2018). Although Image-Guided Radiotherapy (IGRT) with daily imaging can mitigate these toxicities, higher radiation doses continue to pose risks (Wang SL. et al., 2023; Dearnaley et al., 2014; Gharbieh et al., 2025). Placement of a hydrogel spacer into the perirectal fat plane to increase the distance between the prostate and rectum has been developed as a strategy to reduce rectal exposure (Icht et al., 2024). When inserted, the spacer physically displaces the rectum away from the prostate, thereby lowering the rectal radiation dose and reducing toxicity risk (Ohira et al., 2024).

SpaceOAR, an injectable Polyethylene Glycol (PEG) hydrogel, creates this separation and permits SBRT delivery within 24 h of injection, shortening the overall treatment timeline (Saito et al., 2022). In a prospective study of 70 patients treated with SpaceOAR and dose escalation up to 82 Gy, rates of grade 3 gastrointestinal and genitourinary adverse events were 0% and 2.9%, respectively—significantly lower than in prior dose-escalation trials—indicating that hydrogel spacers facilitate safe dose escalation while minimizing rectal toxicity (See et al., 2022). Additional studies have reported superior Planning Target Volume (PTV) coverage in hydrogel-spacer cohorts compared with controls, confirming that spacers allow for higher EBRT doses without compromising rectal sparing (Takayesu et al., 2023; Morita et al., 2024; Kawaguchi et al., 2024). In focal laser ablation, thermal insults to surrounding tissues remain a concern (Alzahrani and Abbas, 2021). Hydrogel spacers, owing to their low thermal conductivity, act as insulating barriers; they have been shown to limit rectal temperatures from 101.1°C to below 42°C during laser ablation, thereby protecting the rectal wall (Namakshenas and Mojra, 2021).

#### 3.1.2 Impact on rectal toxicity and optimization strategies

Hydrogel spacer–induced separation between the prostate and rectum has been shown to correlate inversely with rectal radiation dose and subsequent toxicity. A minimum prostate–rectum distance of 7.5 mm has been identified as the threshold for SpaceOAR efficacy, with greater separation distances yielding reduced rectal exposure (Narukawa et al., 2023). Spacer symmetry has also been demonstrated to positively correlate with both the prostate–PTV distance and the magnitude of rectal dose reduction, thereby enhancing spacer performance (Ohira et al., 2024). however, even asymmetrical spacer distributions significantly decrease rectal dose toxicity (Fischer-Valuck et al., 2017). Spacer thickness varies by anatomical level. One study reported an average hydrogel thickness of 7.1 mm at the prostate apex—significantly less than the 9.4 mm observed at the mid-gland level—resulting in lower mid-gland rectal toxicity (Song et al., 2013). In contrast, Fukumitsu et al. observed greater thickness at the apex with corresponding reductions in rectal radiation exposure (Fukumitsu et al., 2022). These discrepancies are likely attributable to technical variations in injection technique, as the liquid PEG hydrogel distribution is operator-dependent.

In addition, optimization of hydrogel spacer implantation is critical for minimizing rectal toxicity. A retrospective analysis proposed a pacer Quality Score (SQS) system to evaluate hydrogel placement efficacy. Computed Tomography (CT) scans of patients undergoing prostate SBRT demonstrated significant differences in SQS between the anterior and posterior perirectal spaces, with higher total SQS in the anterior gap at the midgland and apex levels; moreover, SQS correlated inversely with rectal dose metrics (Giacometti et al., 2024). A subsequent study employing SQS measurements at the rectal midline and 1 cm lateral to midline confirmed a significant inverse relationship between SQS and late rectal toxicity (Grossman et al., 2023). By applying SQS intraoperatively, implantation quality can be assessed in real time and spacer position adjusted to further reduce rectal exposure. Optimal spacer positioning has been achieved by injecting into the caudal and lateral aspects of the prostate, which further decreases rectal dose (Fukumitsu et al., 2022), and by targeting the midgland plane along the midline (Hwang et al., 2018). Injection technique also influences outcomes: compared with the conventional fixed-needle approach, an apex-directed expansion method yielded greater rectal sparing (Kobayashi et al., 2023). A novel continuous-needle advancement technique—from midgland to apex levels during injection—resulted in significantly increased apex–rectum separation without elevating adverse event rates (Narukawa et al., 2022). Because hydrogel implantation is performed via a perineal approach with limited needle visualization, a disposable, probe-mounted puncture frame has been developed to secure the hydrogel delivery apparatus and enhance real-time visualization of spacer placement around the rectum (Meyer et al., 2021). These advances underscore the importance of refining implantation methods to mitigate radiotherapy-induced rectal toxicity.

#### 3.1.3 Long-term safety and potential adverse effects of hydrogel spacers in PC radiotherapy

The toxicity-reducing benefit of hydrogel spacers during prostate-cancer radiotherapy is well established, but their long-term in-body effects remain uncertain—an issue that may influence some patients’ willingness to choose this adjunctive approach. Extended clinical follow-up is therefore needed to clarify their ultimate value. A 5-year quality-of-life review of PC patients who received hydrogel spacers showed not only durable therapeutic performance but also measurable improvements in overall wellbeing, suggesting a favourable cost–benefit profile (Pinkawa et al., 2017). Nevertheless, hydrogel spacers can still elicit rare adverse events. Hoe et al. reported a case of abscess formation potentially attributable to spacer implantation, and other studies have documented grade-3 complications such as rectal ulceration or fistula (Hoe et al., 2019). Continuous long-term monitoring is thus essential to fully assess the safety profile of hydrogel spacers in clinical practice.

#### 3.1.4 Precision radiotherapy and additional effects

Hydrogel technology offers multifaceted innovations in PC treatment, with its implantable nature enabling unique roles in precision radiotherapy. Under standard departmental practice, patients without contraindications undergo transperineal placement of three Gold Fiducial Markers (GFMs) under local anesthesia to optimize IGRT. However, repeated invasive procedures increase both patient discomfort and economic burden. It has been demonstrated that hydrogels closely mimic the positional stability of intraprostatic GFMs and can serve as real-time anatomical surrogates for IGRT, thereby obviating multiple marker-placement procedures and reducing costs (Icht et al., 2024). To further enhance radiotherapy precision and rectal sparing, an improved polyethylene glycol hydrogel spacer—SpaceOAR Vue™—has been developed. This radiopaque spacer is compatible with Magnetic Resonance Imaging (MRI), thus improving localization accuracy in IGRT while reducing rectal toxicity (Gross et al., 2021).

In moderate hypofractionation protocols, standard hydrogels protect the rectum from radiation injury but degrade slowly over approximately 3 months, with complete absorption possibly extending to 6 months; this prolonged presence may elevate the risk of rare late-onset adverse events (Hamstra et al., 2017). A novel hydrogel formulation has been reported that undergoes rapid degradation following radiotherapy while maintaining excellent imaging conspicuity and biocompatibility throughout treatment (Yu et al., 2023).

Radiation-induced injury to the periprostatic Neurovascular Bundles (NVB) during SBRT is implicated in ED in about 50% of men (Kundu et al., 2004). Hydrogel spacers have been shown to mitigate NVB displacement toward high-dose regions, effectively reducing NVB radiation exposure (Hwang et al., 2021). Although hydrogel spacers lower radiation-induced rectal toxicity, their impact on post-radiotherapy ED remains controversial, potentially reflecting individual variability and limited clinical data (Seymour et al., 2023; Nakai et al., 2024). Further studies are warranted to substantiate these findings.

### 3.2 Applications of hydrogels in bladder cancer immunomodulatory combination therapies

**FIGURE 4:**
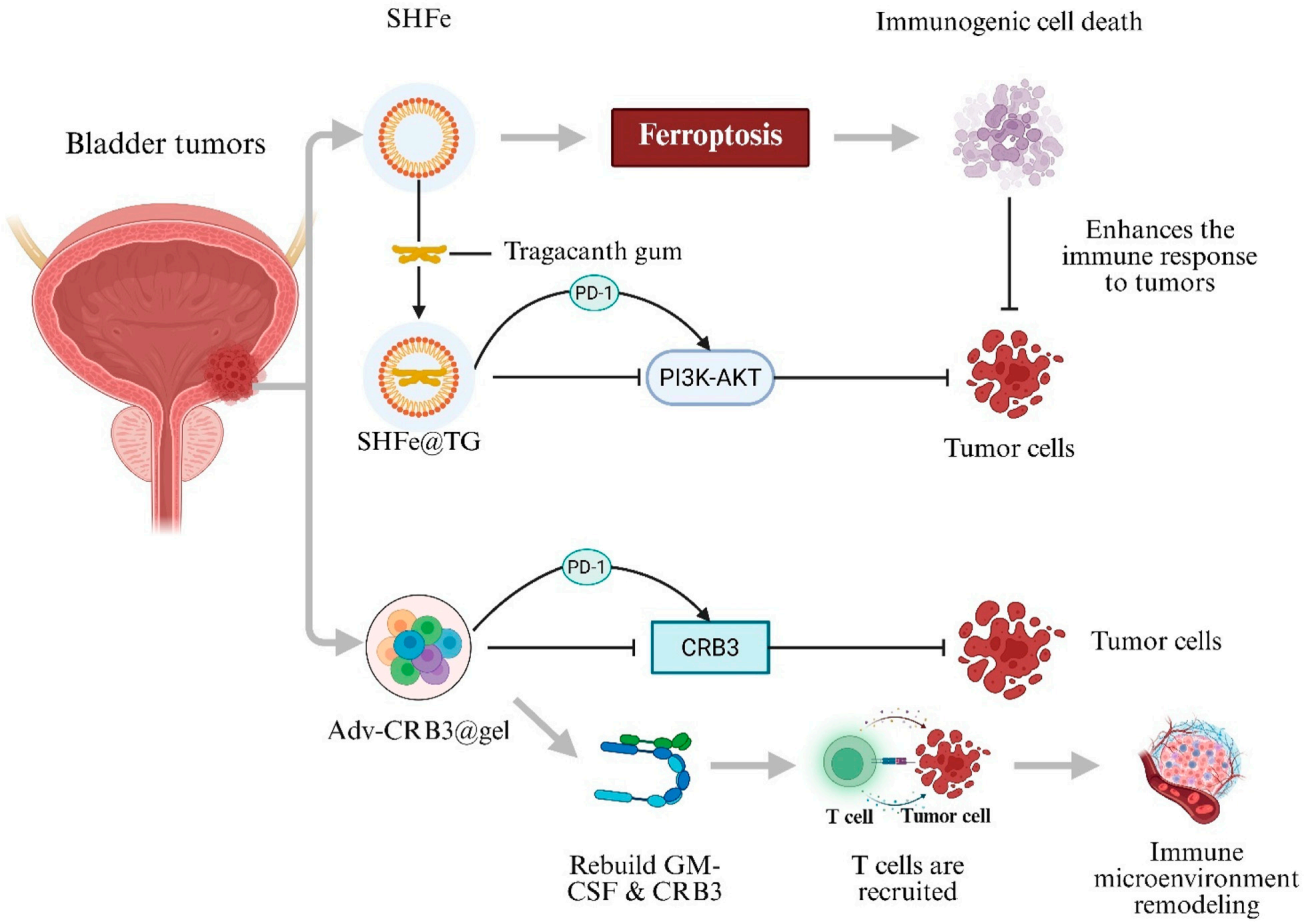
Hydrogel-mediated combination immunotherapy for bladder cancer. Shikonin–Ferric Ion Hydrogel in Tragacanth Gum (SHFe@TG) triggers ferroptosis-induced immunogenic cell death and enhances programmed cell-death protein-1 (PD-1) blockade by inhibiting the PI3K–AKT pathway. Recombinant Oncolytic Adenovirus Hydrogel (Adv-CRB3@gel) Co-Delivers Crumbs-3 (CRB3) and Granulocyte–Macrophage Colony-Stimulating Factor (GM-CSF), recruits T cells and remodels the tumour immune micro-environment.

BC is one of the most common malignancies worldwide and presents significant challenges due to high recurrence rates and substantial burdens on healthcare systems (Lopez-Beltran et al., 2024). BC is classified into non–muscle-invasive BC and muscle-invasive BC. Standard treatments—transurethral resection of bladder tumor and radical cystectomy—are often accompanied by complications such as urinary incontinence and bladder dysfunction, which markedly impair patient quality of life (Alfred et al., 2024). Recently, immunotherapy has emerged as a promising approach for BC treatment. Immune Checkpoint Inhibitors (ICIs), such as programmed cell death protein-1 (PD-1)/programmed death-ligand 1 (PD-L1) antagonists, provide bladder-preserving options by restoring tumor-specific T-cell responses through PD-1/PD-L1 blockade and have demonstrated significant antitumor efficacy (Liu et al., 2024; Rhea et al., 2021). However, ICI monotherapy is limited by high dosage requirements, development of immune tolerance, and immune-related toxicities (Liu et al., 2024). To address these challenges, a Shikonin–Ferric Ion (Fe^3+^) Nanocomposite Hydrogel System (SHFe) was developed to induce ferroptosis-mediated immunogenic cell death. Shikonin encapsulation within SHFe stabilizes the molecule, enhances bioavailability, and enables sustained locoregional delivery. When SHFe is formulated with Tragacanth Gum (SHFe@TG), intravesical drug retention is extended to 72 h. In combination with a PD-1 inhibitor, SHFe@TG synergistically inhibits the Phosphoinositide 3-Kinase–Protein Kinase B (PI3K–AKT) signaling pathway, thereby suppressing BC cell proliferation (Zhao et al., 2025). Furthermore, a Recombinant Oncolytic Adenovirus–Loaded Hydrogel (Adv-CRB3@gel) was engineered to co-deliver an edited CRB3 gene and Granulocyte–Macrophage Colony-Stimulating Factor (GM-CSF). This system not only inhibits BC tumor cells and recruits T cells to remodel the tumor immune microenvironment and enhance tumor antigen–specific immune responses, but it also synergizes with PD-L1 blockade to directly kill BC cells, resulting in marked suppression of tumor proliferation, migration, and invasion (Figure 4) (Zhang WQ. et al., 2024).

### 3.3 Applications of hydrogels in neurovascular repair for erectile dysfunction

ED affects over 150,000,000 men worldwide (Wang CM. et al., 2023). Current ED therapies include oral pharmacotherapy, shockwave therapy, hormone replacement therapy, vacuum erection devices, intracavernosal injections, vascular surgery, and penile prosthesis implantation (Ren Y. et al., 2023). However, these approaches are limited by low bioavailability, systemic adverse effects, poor patient compliance, invasiveness, and narrow applicability (Salonia et al., 2021; Hatzimouratidis et al., 2016; Atipairin et al., 2020). In contrast, hydrogels offer multiple advantages for ED treatment.

**FIGURE 5:**
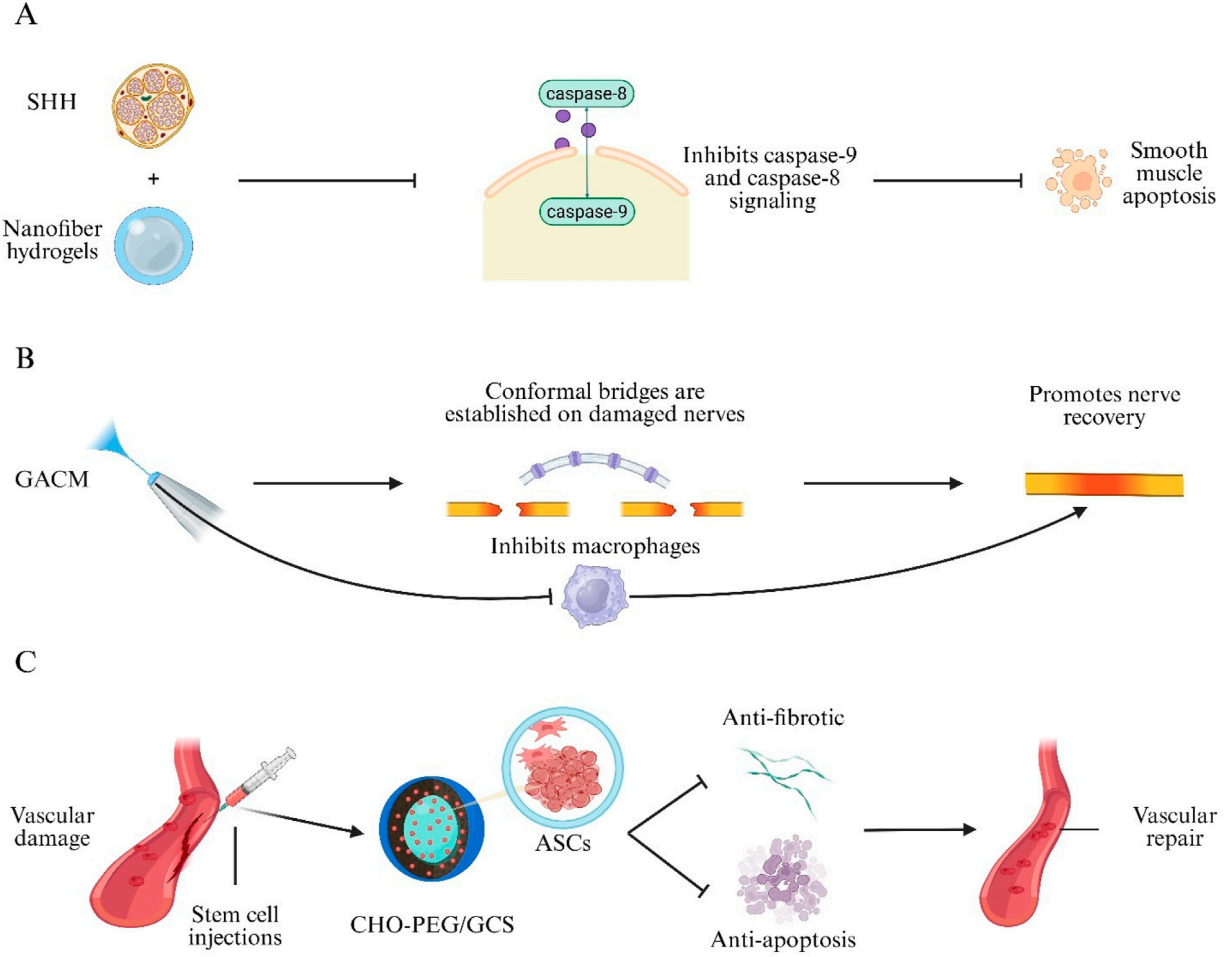
Hydrogel approaches for neurovascular repair in Erectile Dysfunction (ED). **(A)** Sonic-hedgehog peptide-amphiphile nanofibre hydrogel (SHH-PA) blocks caspase-9/-8 signalling, reducing smooth-muscle apoptosis. **(B)** Sprayable Gelatin–Alginate Conductive Matrix (GACM) forms a conformal bridge on damaged cavernous nerves, limits macrophage activity and promotes regeneration. **(C)** Self-healing CHO-terminated poly(ethylene glycol)/glycol-chitosan (CHO-PEG/GCS) hydrogel delivers Adipose-Derived Stem Cells (ASCs) to injured vessels, providing anti-fibrotic and anti-apoptotic cues for vascular repair.

Neurogenic ED results from impaired neural signaling to the corpora cavernosa (Yang et al., 2024). Although phosphodiesterase type 5 inhibitors are first-line agents for ED, they are ineffective in neuronally induced ED (Salonia et al., 2021). Hydrogels have been investigated as biomaterial carriers to promote nerve regeneration and repair nervous system injury associated with cavernosal neuropathy (Bellotti et al., 2021; Nih et al., 2016). A Sonic Hedgehog (SHH)-loaded peptide amphiphile nanofiber hydrogel (SHH-PA) delivers SHH protein and, via a caspase-9-dependent mechanism, inhibits intrinsic caspase-9 and extrinsic caspase-8 signaling, reducing smooth muscle apoptosis and collagen deposition to improve erectile function (Figure 5A) (Martin et al., 2021). Similarly, a sprayable conductive adhesive hydrogel (GACM) with high adhesion and anti-swelling properties conforms to injured nerves to form an electrical bridge, facilitating nerve regeneration and suppressing macrophage-mediated inflammation to restore cavernosal innervation and erectile function (Figure 5B) (Wang et al., 2024).

Erectile Dysfunction in Type 1 Diabetes Mellitus (T1D-ED) is a common complication of diabetes mellitus (Wessells et al., 2018). Stem cell therapies have been explored for T1D-ED but are hindered by hemodynamic washout of cells into cavernosal venules, especially in the flaccid state (Aziz et al., 2010; Huang et al., 2010). To address this, a self-healing hydrogel composed of benzaldehyde-terminated PEG and Glycol Chitosan (GCS) (CHO-PEG/GCS) was engineered to encapsulate Adipose-Derived Stem Cells (ASCs), prolong cellular retention, and synergize angiogenic, antifibrotic, and antiapoptotic effects to restore erectile function in diabetic ED models (Figure 5C) (Yan et al., 2020). These studies demonstrate the broad potential of hydrogels for neurovascular repair in ED.

### 3.4 Applications of hydrogels in male reproductive medicine

In recent years, hydrogel technologies have demonstrated significant potential in male infertility treatment and reproductive tissue engineering due to their biomimetic properties and tunable physicochemical characteristics, particularly in sperm selection and release and in testicular tissue transplantation and regeneration. Male-factor infertility accounts for 30%–50% of infertility cases and has increased globally (Eisenberg et al., 2023). Assisted reproductive technologies, including Intracytoplasmic Sperm Injection (ICSI), have markedly improved pregnancy rates (Esteves et al., 2018). However, ICSI bypasses the natural sperm selection mechanisms of the female reproductive tract and the oocyte’s zona pellucida barrier (Sánchez-Calabuig et al., 2014), Defective sperm can lower fertilisation rates, impair embryo quality and markedly raise the risk of miscarriage, thereby greatly increasing the likelihood of ART failure and potentially posing unknown long-term health risks to offspring (Li et al., 2023). Consequently, selecting high-quality sperm has become a pivotal challenge.

**FIGURE 6:**
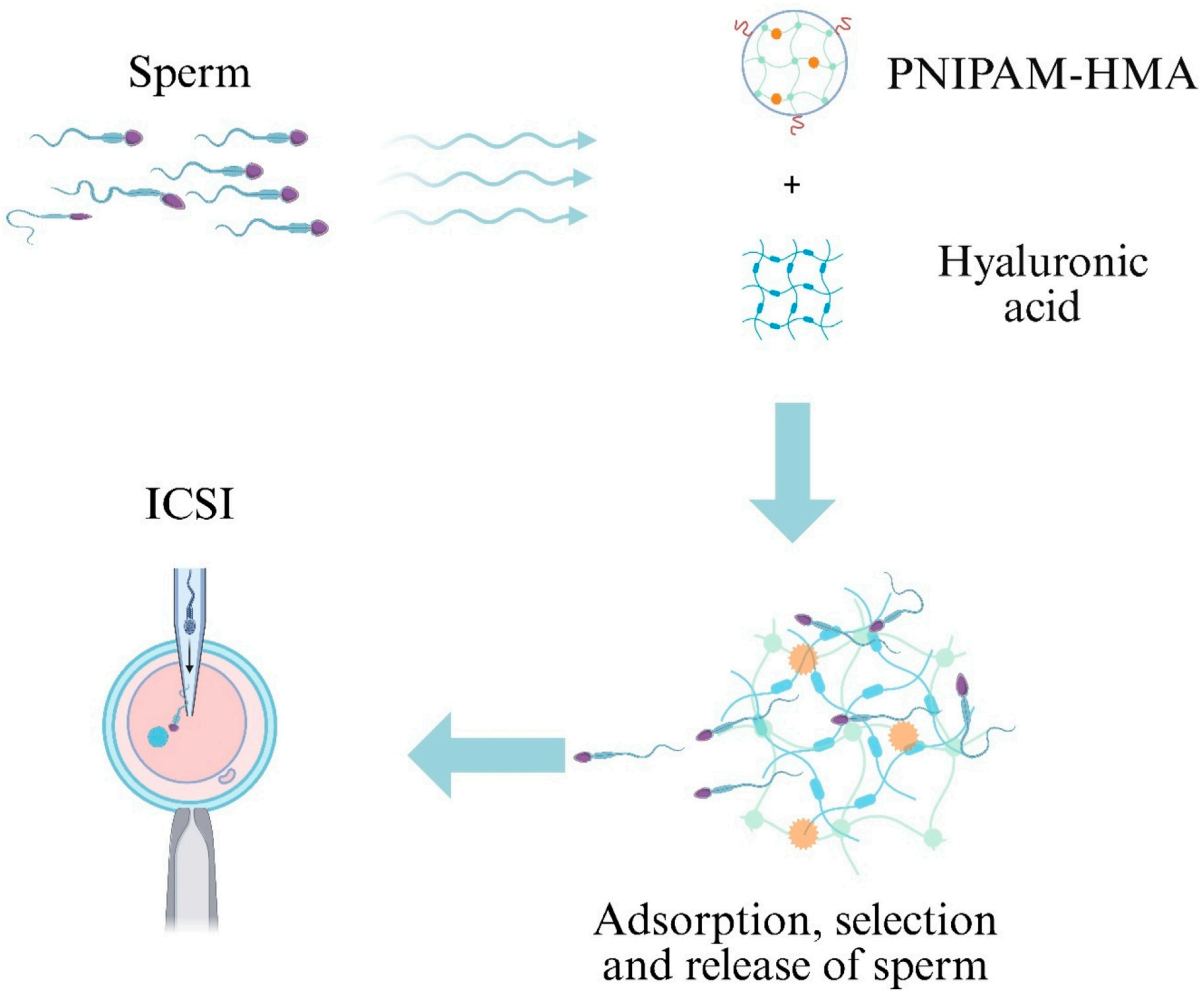
Hydrogel-assisted sperm selection for Intracytoplasmic Sperm Injection (ICSI).

HA, a natural polymer synthesized by various cell types in reproductive and nonreproductive tissues, has been shown to selectively bind sperm with high motility and intact acrosomes, thereby improving ICSI outcomes (Casillas et al., 2018; Canepa et al., 2019). A semi-interpenetrating polymer network hydrogel composed of poly(N-isopropylacrylamide-co-hydroxyethyl methacrylate) (PNIPAM-HMA) and HA was developed to achieve charge-mediated sperm capture. In bovine models, this hydrogel achieved 50% specific attachment of bull spermatozoa. Following hyaluronidase treatment, 47% of bound sperm were released; these released sperm exhibited 70% progressive motility and maintained high plasma-membrane integrity (Figure 6) (Blois et al., 2021).

Testicular tissue autotransplantation and *in vitro* maturation techniques have garnered significant attention, particularly for prepubertal pediatric oncology patients who do not produce sperm and whose future fertility may be irreversibly compromised by chemotherapy and radiotherapy (Wyns et al., 2011; Wyns et al., 2015). However, limitations in current cryopreservation protocols predispose grafts to ischemic injury during revascularization, substantially reducing spermatogonial survival and viability posttransplantation (Poels et al., 2013; Van Saen et al., 2013). The spatiotemporal delivery of Vascular Endothelial Growth Factor (VEGF) is a critical determinant of graft outcome (Cao et al., 2005). In murine models, alginate hydrogels loaded with testicular tissue and VEGF were histologically analyzed posttransplantation; VEGF-laden grafts exhibited significantly increased microvascular density and reduced undifferentiated spermatogonial counts, demonstrating that alginate hydrogels effectively preserve spermatogonia and enhance posttransplant recovery of testicular architecture (Poels et al., 2016).

Spermatogenesis—the proliferation and differentiation of Spermatogonial Stem Cells (SSCs) within seminiferous tubules—requires efficient culture systems for fertility preservation and assisted reproductive technology applications (Stukenborg et al., 2008; Zhang J-Y. et al., 2024). Conventional SSC culture relies on feeder-layer cells, resulting in inefficiencies and contamination risks (Yang et al., 2020). Decellularized Testicular Matrix (DTM) hydrogels have emerged as promising scaffolds for generating testicular organoids (Saldin et al., 2017). A three-dimensional (3D) culture system utilizing DTM hydrogel was developed and seeded with murine SSCs. Compared with feeder-layer methods, SSC proliferation rates were markedly increased, and gene-expression profiles matched those of native SSCs, offering a feeder-free approach to more efficient *in vitro* SSC expansion and differentiation (Yang et al., 2020). Furthermore, the incorporation of epididymal cells and glial cell line–derived neurotrophic factor into the DTM hydrogel system was shown to enhance postthaw SSC proliferation. This composite system significantly reduced caspase-3 expression and upregulated miR-21, resulting in increased spermatogenic cell numbers, indicative of antiapoptotic effects and enhanced SSC regenerative capacity (Rahbar et al., 2024).

A semi-interpenetrating poly(N-isopropylacrylamide-co-hydroxyethyl methacrylate)/hyaluronic-acid (PNIPAM-HMA/HA) network captures motile, acrosome-intact sperm via charge interactions; brief hyaluronidase treatment then releases the enriched sperm population for ICSI.

### 3.5 Applications of hydrogels in urinary tract reconstruction

Hydrogels formulated with biomaterials such as alginate, Silk Fibroin (SF), and Bladder Acellular Matrix (BAM) exhibit exceptional biocompatibility, tunable physicochemical characteristics, and cell‐adhesive and–differentiative capacities, conferring high stability even in hostile environments. Consequently, they hold significant promise for bladder and urethral repair and reconstruction.

#### 3.5.1 Applications in bladder tissue reconstruction

The incidence of bladder disorders—including BC, bladder contracture, congenital malformations, and trauma—has increased, leading to an increased need for surgical bladder repair and reconstruction (Shi et al., 2017). Current reconstruction methods are limited by risks of infection, metabolic disturbances, and secondary malignancies (Lam Van Ba et al., 2015). Effective bladder reconstruction requires scaffolds with adequate mechanical strength and a biomimetic microenvironment (Xiao et al., 2017). Hydrogels have been adopted as key materials in bladder tissue engineering due to their favorable properties.

BAM–based scaffolds combine robust mechanical performance with endogenous growth factors such as Vascular Endothelial Growth Factor (VEGF) and Patelet-Derived Growth Factor-BB (PDGF-BB). SF enhances mechanical integrity and provides a biomimetic surface for cell adhesion and differentiation (Seth et al., 2013; Jiang et al., 2017). In one study, an SF solution was processed into a bilayer silk film–sponge scaffold and combined with pepsin-digested BAM to form a hydrogel composite. This system exhibited excellent retention and stability of VEGF and PDGF-BB and promoted smooth muscle regeneration and angiogenesis, highlighting its potential for bladder reconstruction (Xiao et al., 2021).

Although natural hydrogels offer favorable biological properties, their mechanical strength may be insufficient for bladder tissue regeneration. Synthetic hydrogels can be modified to enhance mechanical properties but often lack protein- and cell-adhesive sites (Fu et al., 2023). To address this, a composite hydrogel composed of acrylic ester polymers, extracellular matrix components, and bladder smooth muscle cells was developed. This hydrogel significantly improved mechanical strength and elastic modulus while maintaining strong capacity for bladder tissue repair and regeneration (Sivaraman et al., 2015).

#### 3.5.2 Applications in urethral reconstruction

Severe urethral strictures commonly arise following major trauma, inflammation, malignancy, or congenital anomalies (Xu et al., 2009). Strictures can lead to ED, painful erections, urethral fistulae, and stone formation (Chapple et al., 2014). However, limited availability of autologous graft tissue, the harsh UME, and deficient scarless‐repair mechanisms often result in repair failure (Mangir et al., 2019). In tissue‐engineering approaches, ECM–based scaffolds such as BAM and SF have shown promise. In a New Zealand rabbit model of urethral defect, a bilayer hydrogel scaffold composed of porous BAM hydrogel and dense SF was implanted. The scaffold markedly increased neovascularization and maintained a patent urethral caliber. Histological analysis revealed formation of a continuous, multilayered urothelial lining and aligned smooth‐muscle regeneration, demonstrating support for both angiogenesis and tissue regeneration (Cao et al., 2019).

To overcome the UME barrier—which impedes materials, promotes fibrosis, and fails to direct progenitor differentiation—an UME‐responsive composite hydrogel (SA-nHA-SiQD) was fabricated using layer-by-layer three-dimensional bioprinting. This construct combines a sodium alginate backbone with Silicon Quantum Dots (SiQDs) to enable structural remodeling under UME conditions, inhibit Transforming Growth Factor-β/smad (TGF-β/Smad) signaling, and activate Matrix Metalloproteinase-1 (MMP-1) and Collagen Type III Alpha One Chain (COL3A1). These effects promote epithelial proliferation and differentiation, modulate collagen deposition, reduce fibrosis, and generate Reactive Oxygen Species (ROS) to enhance scaffold strength—thereby enabling scarless, “memory” repair of the urethra (Mao et al., 2020; Yang M. et al., 2022). Additionally, electrospun Polyurethane-Urea (PUU) fiber membranes coated with collagen (cPUU) were developed as stretchable hydrogel scaffolds. These cPUU scaffolds exhibited a tensile elongation at break of 404% ± 40% and supported adhesion and proliferation of bladder smooth‐muscle cells *in vitro*. *In vivo* evaluation demonstrated that cPUU hydrogels significantly promoted re‐epithelialization of urethral defects and facilitated effective urethral repair (Wang et al., 2019).

### 3.6 Applications of hydrogels in male urogenital tract infections

Chronic inflammation of the bladder and prostate remains inadequately managed by current therapies. Novel hydrogel systems, formulated via diverse crosslinking strategies, offer sustained drug release, enhanced inflammatory repair, and improved barrier penetration, providing efficient treatment avenues for chronic urogenital inflammation.

Interstitial Cystitis/Bladder Pain Syndrome (IC/BPS) is characterized by pelvic pain, lower urinary tract symptoms, and bladder hypersensitivity (Payne et al., 2007). Intravesical injection of OnabotulinumtoxinA (BTX-A) is an established therapy; however, the injection procedure can be extremely painful for IC/BPS patients and is associated with bleeding, infection, and urinary retention (Rappaport et al., 2018). Intravesical instillation has emerged as a less invasive alternative but is hampered by rapid drug washout and potential instillation-related adverse events (Krhut and Zvara, 2011). A temperature-responsive TC-3 hydrogel system (TC-3) was developed to deliver BTX-A via bladder instillation. Following treatment, patients exhibited significant reductions in pain visual analog scale scores, inflammatory index, problem index, and nocturia frequency at week 6. These findings indicate that the TC-3 hydrogel enables continuous intravesical BTX-A release while mitigating instillation-related side effects (Rappaport et al., 2018).

Hemorrhagic Cystitis (HC) is a debilitating inflammatory condition of the bladder characterized by hemorrhage and ulceration, often accompanied by severe pain, hematuria, and dysuria, which profoundly impair patient quality of life (D'Amico et al., 2022). HC can be classified into subtypes, with chemical HC typically induced by chemotherapeutic agents such as cyclophosphamide (Matz and Hsieh, 2017). Current treatments—including pharmacotherapy and surgical intervention—are limited by systemic side effects and risks of infection, anesthesia‐related complications, and prolonged recovery, respectively (Payne et al., 2013). A major challenge is maintaining robust adhesion and mechanical integrity of intravesical therapies in the bladder’s dynamic fluid environment under cyclic mechanical stresses. To address these obstacles, a Silk Fibroin Methacryloyl (SFMA)/tea polyphenol composite hydrogel was engineered via photopolymerization. This hydrogel exhibits strong mucoadhesive strength and excellent compressive resilience, enabling it to withstand cyclic bladder distension. In a cyclophosphamide‐induced rat HC model, SFMA/tea polyphenol hydrogels significantly reduced hematuria, suppressed interleukin-1β (IL-1β) expression, and upregulated CD31, CD63, and α-smooth muscle actin (α-SMA), thereby enhancing neovascularization and smooth muscle regeneration and accelerating tissue repair (Chen et al., 2024). Similarly, a Chitosan Methacryloyl (CHMA)/SFMA hydrogel system—produced via photoinitiated crosslinking and methacrylation—demonstrated high adhesive strength and rapid hemostasis on injured porcine bladder tissue. *In vitro*, the CHMA/SFMA hydrogel promoted microaggregate formation of ureteral epithelial cells and modulated macrophage polarization, facilitating inflammation resolution, accelerating angiogenesis and myogenesis, and improving HC-associated bladder damage (Yao et al., 2024).

Chronic Prostatitis (CP) is a syndrome characterized by pelvic pain and lower urinary tract symptoms (Rees et al., 2015). More than 90% of CP cases are classified as Chronic Nonbacterial Prostatitis (CNP) (Cyril et al., 2022). Current treatments for CNP include antibiotics, alpha-adrenergic blockers, and nonsteroidal anti-inflammatory drugs (Franco et al., 2019). Emodin (EMO), a natural anthraquinone derivative, exhibits anti-inflammatory, antioxidant, and antifibrotic properties; however, its therapeutic efficacy is limited by poor permeability across the Blood–Prostate Barrier (BPB) (Xia et al., 2021). To overcome this barrier, a triple-targeted, thermosensitive rectal hydrogel nanodelivery system was engineered by dialysis and electrostatic adsorption to encapsulate EMO. This system targets lactoferrin receptors on intestinal epithelial NCM460 cells and prostate epithelial RWPE-1 cells, as well as CD44 receptors on macrophages, thereby significantly enhancing cellular uptake and inhibiting Toll-Like Receptor 4/Nuclear Factor-κB (TLR4/NF-κB) signaling. Furthermore, the hydrogel penetrates the BPB and accumulates in the prostatic acini, resulting in marked reductions in inflammatory cytokines, oxidative stress markers, and fibrosis in a CNP rat model, all without significant systemic toxicity (Ye et al., 2024).

### 3.7 Applications of hydrogels in other andrological disorders

Hydrogel materials have been extensively investigated across major andrological fields—PC, BC, ED, reproductive medicine, urinary tract reconstruction, and genitourinary infections—and have also shown potential in treating Overactive Bladder (OAB).

OAB, which is characterized by detrusor smooth muscle overactivity leading to increased urinary frequency, urgency, and occasional incontinence, is conventionally managed with anticholinergic agents (Wein, 2003). Oxybutynin Hydrochloride (Oxy) reduces bladder contractility by blocking postganglionic muscarinic receptors, thereby lowering intravesical pressure and raising the micturition threshold; however, long-term oral administration is limited by systemic side effects (Escobar et al., 2021). To address these limitations, a composite hydrogel was engineered via dynamic Schiff-base crosslinking between aldehyde-functionalized oxidized dextran and carboxymethyl chitosan to encapsulate Oxy, enabling localized sustained release. In OAB rat models, intravesical administration of the Oxy-loaded hydrogel was found to significantly reduce systemic adverse effects, downregulate Orai1/STIM1 calcium-channel proteins, and improve urodynamic parameters, demonstrating a promising targeted drug-delivery strategy for OAB therapy (Sun et al., 2022).

## 4 Applications of hydrogels in andrological disease diagnostics

Hydrogel‐based diagnostic platforms have been investigated primarily for PC and BC, reflecting the high mortality rates and profound quality-of-life impact of these malignancies, as well as limitations in current diagnostic modalities (Phookum et al., 2024). Research has focused on leveraging hydrogel technologies to develop more streamlined, sensitive, and efficient diagnostic assays.

### 4.1 Applications in prostate cancer diagnostics

**FIGURE 7:**
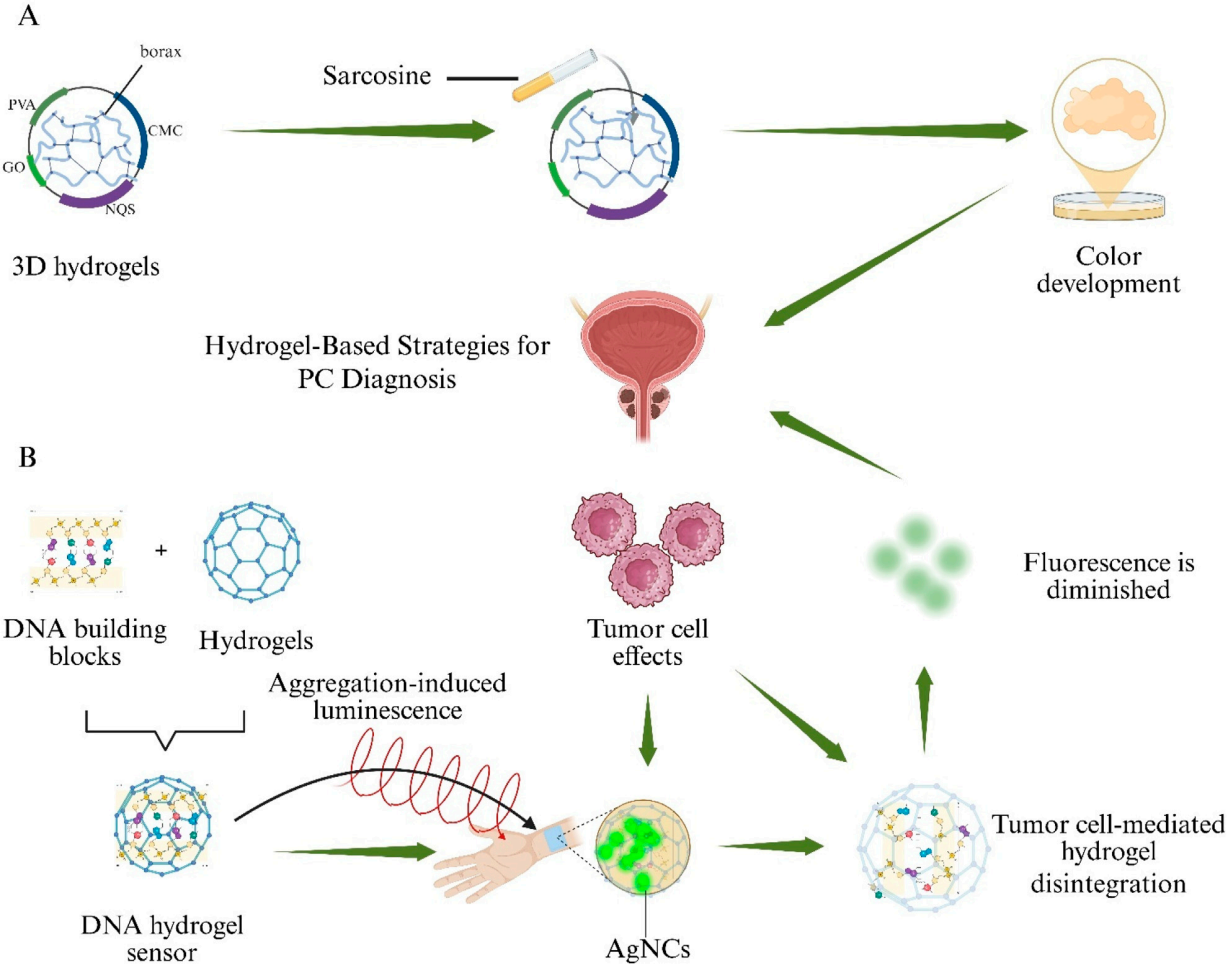
Hydrogel-based sensing systems for non-invasive Prostate Cancer (PC) detection. **(A)** A borax-cross-linked PVA/CMC/GO/NQS hydrogel produces a visible colour change in the presence of urinary sarcosine. **(B)** A DNA hydrogel aptasensor loaded with aggregation-induced-emission silver nanoclusters (AIE-AgNCs) collapses upon binding Prostate-Specific Antigen (PSA), resulting in fluorescence quenching.

PC 5-year and 10-year survival rates are estimated at 65%–90% for patients diagnosed at an early stage, making early detection and accurate identification of PC critical for selecting optimal treatment strategies (Damborska et al., 2017). Although diagnostic methods such as Enzyme-Linked Immunosorbent Assay (ELISA), Surface Plasmon Resonance Imaging (SPRI), and PSA testing are available, they remain invasive, time-consuming, costly, and procedurally complex (Liu et al., 2016). Consequently, a noninvasive assay is urgently needed to replace these traditional approaches. Elevated urinary sarcosine levels have emerged as a novel biomarker of PC cell growth (Sreekumar et al., 2009). A 3D hydrogel was fabricated by borax cross-linking regenerated cellulose (sourced from waste paper) with PVA, Carboxymethyl Cellulose (CMC), Graphene Oxide (GO), and 2-naphthoquinone-4-sulfonic acid sodium salt (NQS). This PVA/CMC/GO/NQS hydrogel exhibits a linear colorimetric response to sarcosine over a 0–100 µM range, shows negligible interference from urea or uric acid, and produces urinary assay results that correlate closely with mass spectrometry analyses, demonstrating its potential for noninvasive PC screening (Figure 7A) (Phookum et al., 2024).

A DNA-based hydrogel aptasensor employing Silver Nanoclusters with Aggregation-Induced Emission (AIE–AgNCs) was prepared to detect Prostate-Specific Antigen (PSA) without antibodies. Upon PSA binding, hydrogel collapse occurred and fluorescence intensity decreased proportionally, enabling quantification down to 4.4 pg mL^−1^. The sensor exhibited minimal cross-reactivity with Immunoglobulin G (IgG) and provided a rapid, highly sensitive alternative to conventional immunoassays (Figure 7B) (Ahmadi-Sangachin et al., 2023).

PSA is a single-chain glycoprotein secreted by normal and malignant prostatic cells, and its serum levels are increased in PC (Meyer and Gorin, 2019). Although serum PSA concentration is widely utilized as a diagnostic biomarker for PC, it may lead to overdiagnosis and unnecessary prostate biopsies (Barry, 2001). Therefore, improved noninvasive and highly specific diagnostic methods are required. Detection of PC-associated biomarkers in bodily fluids such as urine is an attractive approach (Alix-Panabier̀es and Pantel, 2013). Exosomal microRNAs (miRNAs) have emerged as promising biomarkers for PC (Gao et al., 2019). A hydrogel-based HCR assay was developed for the detection of urinary exosomal miRNAs and validated clinically, demonstrating high sensitivity and specificity. This method quantified two urinary exosomal miRNAs—hsa-miR-6090 and hsa-miR-3665—with approximately 35-fold signal amplification and attomole-level limits of detection, indicating its potential as an adjunctive diagnostic tool for PC (Kim et al., 2021).

### 4.2 Applications in bladder cancer diagnostics

BC affects approximately 550,000 new cases annually and ranks among the top ten most common malignancies worldwide, imposing a severe burden on patient quality of life (Richters et al., 2020). Early detection and treatment of BC have been shown to achieve high cure rates (Kamat et al., 2016). Cystoscopy, CT, and MRI are the principal methods for early BC diagnosis; however, their invasiveness and ionizing‐radiation exposure limit routine use (Karaoglu et al., 2014).

**FIGURE 8:**
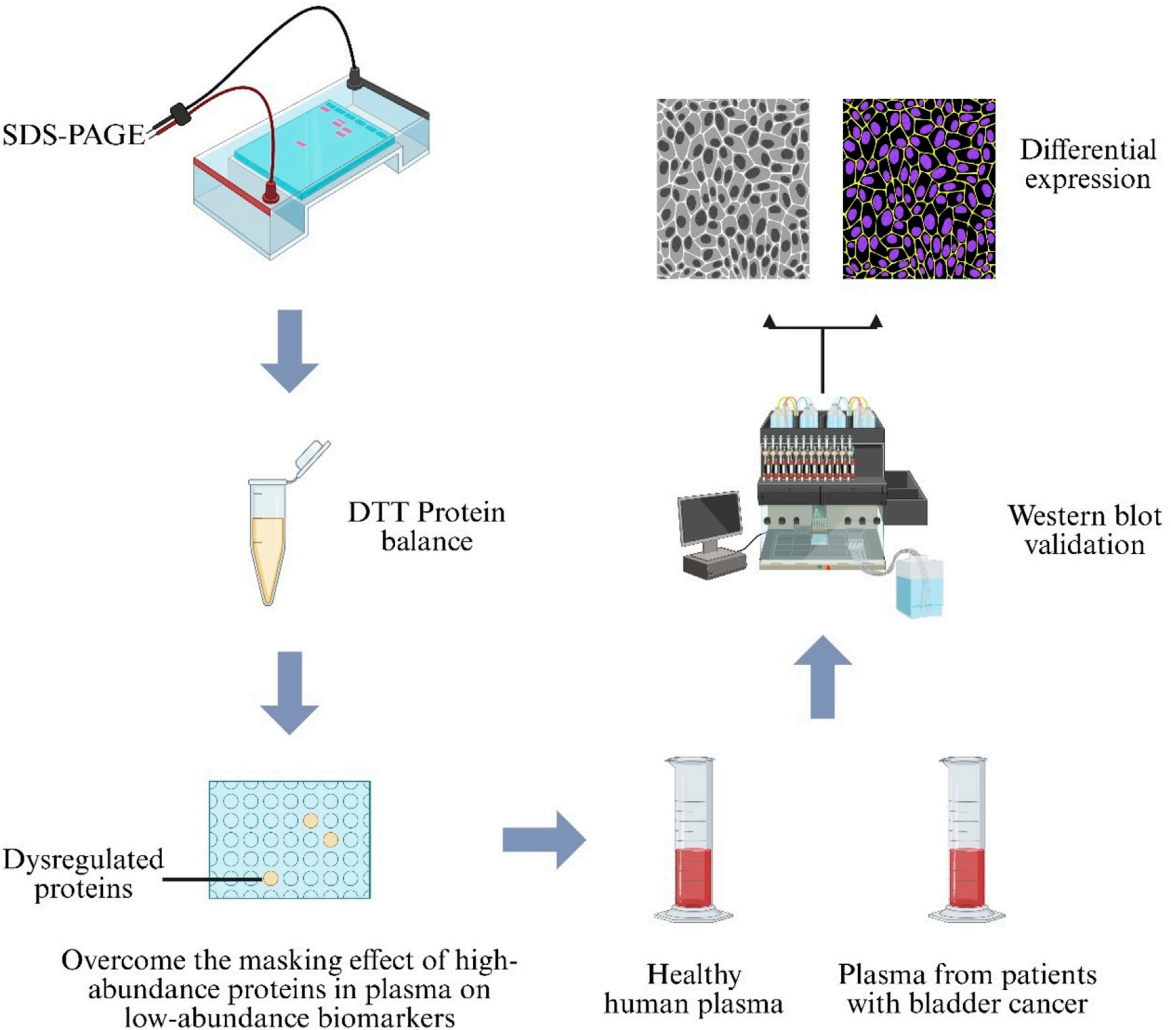
Proteomic workflow for bladder-cancer biomarker discovery. Plasma from healthy donors and Bladder Cancer (BC) patients is first treated with Dithiothreitol (DTT) to balance protein abundance, then separated by Sodium-Dodecyl-Sulfate Polyacrylamide-Gel Electrophoresis (SDS-PAGE). Differentially expressed bands are confirmed by Western blot analysis.

Plasma‐based proteomic biomarker discovery is hindered by masking effects of high‐abundance proteins, and current depletion strategies are costly and time‐consuming (Anderson and Anderson, 2002). To address this, a protein‐equalization technique using Dithiothreitol (DTT) was coupled with Two‐Dimensional Sodium Dodecyl Sulfate–Polyacrylamide Gel Electrophoresis (2D‐SDS‐PAGE). Analysis of BC patient sera revealed overexpression of six proteins—albumin, gelsolin, fibrinogen γ chain, immunoglobulin α-1 chain C region, immunoglobulin α-2 chain C region, and haptoglobin—which were proposed as BC-associated biomarkers, offering a rapid, low-cost marker‐discovery strategy (Figure 8) (Araújo et al., 2018).

Optical fluorescence imaging within the second near-infrared window (NIR-II; 1,000–1,700 nm) has improved spatial resolution and tissue penetration for early tumor detection (Liu et al., 2022). However, NIR-II diagnostics require sufficient nanoprobe accumulation, and short bladder retention times limit effectiveness (Yoon et al., 2020). To overcome these challenges, an ultraminiature AuPd–Poly(Amidoamine)–Folic Acid (AuPd-P-FA) nanoprobe was incorporated into a temperature-responsive poly(amidoamine) hydrogel for bidirectional intravesical infusion. Folic acid targeting enhanced tumor-specific binding, shifted fluorescence emission to 1,250 nm, and increased quantum yield, thereby prolonging bladder residence and enriching signal in BC lesions—offering a novel noninvasive diagnostic strategy for BC (Ren SL. et al., 2023).

## 5 Applications of hydrogels in andrological drug delivery

Hydrogel systems have been shown to hold considerable promise for drug delivery in andrological applications. By leveraging their intrinsic properties and optimizing fabrication processes, these systems can prolong drug residence time, increase local drug concentration, and—through thermosensitivity and other stimuli-responsive features—provide more effective therapeutic options for andrological diseases.

### 5.1 PC drug delivery

In drug-delivery contexts, smart responsive hydrogels address limitations of conventional chemotherapy (Table 2). DTX is a chemotherapeutic agent that induces apoptosis via phosphorylation of B-cell lymphoma 2 (Bcl-2) to inhibit tumour growth; however, its high lipophilicity and low aqueous solubility complicate its delivery (Shekari et al., 2025). Similarly, Doxorubicin (DOX) suffers from poor water solubility and systemic toxicity (Harris et al., 2002). To overcome these challenges, a star-shaped peptide supramolecular hydrogel responsive to zinc ions was developed. *In situ* self-assembly is triggered by the high zinc-ion concentration in prostatic tissue, enabling targeted DTX delivery and sustained release, and proliferation of DU-145 and PC-3 cells was inhibited in a dose-dependent manner (Tao et al., 2019). Silveira et al. formulated a composite thermosensitive hydrogel embedding DOX-loaded Mesoporous Silica Nanoparticles (MSNs). Using Pluronic F127s body-temperature–induced gelation, this intraperitoneal depot selectively adsorbs DOX within its nanopores and releases it via electrostatic interactions, resulting in a significant reduction in invasive PC incidence and decreased myocardial uptake of DOX (Silveira et al., 2016). These studies demonstrate the potential of hydrogel platforms to optimize PC therapy through targeted delivery and local controlled release.

**TABLE 2:** Applications of hydrogel systems in drug delivery.

Category	Drug Loaded	Limitations of Free Drug	Formulation and Features	Function	Target Disease	References
Supramolecular Hydrogel	DTX	High lipophilicity and low solubility	Zinc‐ion–responsive star‐shaped peptide hydrogel self‐assembled *in situ* by prostatic Zn2+ triggers	Targeted DTX delivery and sustained release; dose‐dependent inhibition of DU‐145 and PC-3 cell growth	PC	Tao et al. (2019)
	DOX	Poor water solubility; systemic toxicity	Semi‐interpenetrating CCA hydrogel (chitosan + SBE-β-CD + CD)	Prolonged intravesical DOX retention	Urothelial carcinoma	Zhang et al. (2024c)
	EPO	Inefficient *in vivo* transport	Chitosan/alginate hydrogel for local EPO encapsulation	↑ Sperm motility; ↓ testicular malondialdehyde levels	Male infertility	Rasekh et al. (2025)
Thermosensitive Hydrogel	DOX	Poor water solubility; systemic toxicity	DOX‐loaded mesoporous silica nanoparticles + Pluronic F127 gel depot	Extended DOX release; ↓ invasive PC incidence; ↓ myocardial DOX uptake	PC	Silveira et al. (2016)
	Que	Poor aqueous solubility	P407 thermosensitive *in situ* gel with Que‐loaded lipid vehicles	Extended Que retention *ex vivo*; ↓ T-24 cell viability	Urothelial carcinoma	Shawky et al. (2022)
	RAP	Poor solubility, permeability, and uptake	RAP‐loaded liposomes (±FA‐modification) dispersed in P407 thermogel	2× ↑ RAP cellular uptake; 60% ↑ tumor inhibition	Urothelial carcinoma	Yoon et al. (2019)
	ASC-Exo	Rapid clearance from corpora cavernosa	In situ–polymerized polydopamine nanoparticle–PELA thermogel; photoacoustic‐guided injection	Precise, sustained ASC-Exo release to tunica albuginea	ED	Liang et al. (2022b)
	atRA	Poor solubility, instability, rapid metabolism	Thermosensitive CS/β-glycerophosphate hydrogel scaffold	Prevents atRA degradation; promotes spermatogonial differentiation, meiosis, sperm release; ↑ *in vivo* atRA dose	Male infertility	Zarei et al. (2025)
*In Situ* Gel–Liposome (LP-Gel)	Paclitaxel	Rapid urinary clearance; poor urothelial penetration/adhesion	Fluidic liposomes embedded in gellan‐gum hydrogel; ion‐triggered cross‐linking	↑ urothelial permeability; prolonged paclitaxel bladder retention	Urothelial carcinoma	GuhaSarkar et al. (2017)
Microemulsion Hydrogel	Sildenafil citrate	Low bioavailability; systemic side effects; delayed onset	Non‐microemulsion hydrogel with isopropyl myristate and water	1.97× ↑ membrane permeation; ↓ metabolites by 50%; faster onset	ED	Atipairin et al. (2020)
Bigels	Que	High‐dose oxidative toxicity	Cottonseed/hempseed oil‐based bigel with acacia gum	↓ Que oxidative toxicity; ↓ sperm abnormalities and DNA fragmentation; ↑ serum testosterone	Male infertility	Mousavi et al. (2021)

### 5.2 Drug delivery in urothelial carcinoma

Urothelial carcinoma ranks among the ten most common malignancies worldwide, with BC as the predominant subtype (Sung et al., 2021). Although therapeutic advances have markedly reduced mortality, postoperative recurrence remains a major clinical challenge (Rouprêt et al., 2023). Intravesical chemotherapy is recognized as an effective strategy to lower recurrence rates; however, its efficacy is hampered by the urothelial barrier, short drug residence time, and suboptimal targeting (GuhaSarkar et al., 2017). To address these limitations, a biodegradable *in situ* Gel–Liposome Hydrogel (LP-Gel) system was developed for bladder instillation. In this system, fluidic liposomes are embedded within a gellan gum hydrogel matrix and ion-triggered to form a cross-linked network, thereby enhancing permeability across the urothelial barrier and adhesion to the mucin layer. As a result, paclitaxel retention in the bladder was significantly prolonged (GuhaSarkar et al., 2017). Additionally, a polysaccharide-based supramolecular injectable hydrogel, termed Chitosan–Cyclodextrin Amphiphilic (CCA), was engineered via one-step co-encapsulation of CS, Sulfobutyl ether-β-Cyclodextrin (SBE-β-CD), and β-Cyclodextrin (β-CD) with DOX. In a murine BC model, this CCA hydrogel extended intravesical DOX retention and markedly enhanced its inhibitory effect on bladder tumour cells (Zhang C. et al., 2024).

Quercetin (Que), a natural polyphenolic flavonoid with potent anticancer activity and low toxicity, has been widely investigated for BC therapy. However, its poor aqueous solubility substantially reduces intravesical residence time (Pralhad and Rajendrakumar, 2004). To overcome this limitation, carrier systems were employed to enhance delivery and retention of Que. Shawky et al. evaluated various lipid vehicles for Que solubilization and used Poloxamer 407 (P407) as a thermosensitive *in situ* gel to create a mucoadhesive depot. This P407 formulation achieved Que penetration depths of up to 350 µm in *ex vivo* bladder tissue, extended retention to 24 h, and significantly inhibited T-24 cell viability (Shawky et al., 2022). Similarly, Rapamycin (RAP) was incorporated into both unmodified and folic acid–modified liposomes via thin-film hydration and preloading, and these liposomes were dispersed in a P407 thermosensitive hydrogel. This system doubled RAP cellular uptake and, through erosion-controlled release, increased tumor-inhibition rates by 60% in BC models (Yoon et al., 2019).

Intravesical instillation remains a cornerstone of BC treatment, yet most instilled drug is expelled during the first void, and the procedure can provoke urinary tract obstruction (Collado et al., 2000). To address these challenges, a floating hydrogel system was developed by generating microbubbles within the gel to reduce its density, allowing it to float atop urine. When hydrogel density falls below that of urine, buoyancy is maintained, thereby markedly reducing the risk of urinary tract blockage (Lin et al., 2016).

### 5.3 Drug delivery in ED

Sildenafil citrate is widely used to treat mild‐to‐moderate ED; however, it exhibits low oral bioavailability, frequent systemic adverse effects, and delayed onset of action (Elnaggar et al., 2011). A non-microemulsion hydrogel was formulated by incorporating isopropyl myristate and water with sildenafil citrate, resulting in a membrane permeation rate 1.97-fold higher than that of the pure drug solution, a 50% reduction in metabolite formation, and a shortened onset time (Atipairin et al., 2020). Intramuscular administration of Adipose-Derived Stem Cell Exosomes (ASC-Exo) has emerged as a promising ED therapy; however, the high vascularization of the corpora cavernosa limits ASC-Exo retention (Liang L. et al., 2022). To overcome this, an in situ–Polymerized Polydopamine Nanoparticle–Poly(Ethylene Glycol)–Poly(Lactic Acid) Thermosensitive Hydrogel (PDNPs–PELA) was developed and, when combined with photoacoustic imaging guidance, enabled precise, sustained delivery of ASC-Exo to the tunica albuginea, providing a novel approach to ED treatment (Liang L. et al., 2022).

### 5.4 Drug delivery in male infertility

All-Trans Retinoic Acid (atRA), an active metabolite of vitamin A, supports blood–testis barrier formation and promotes spermatogonial differentiation (Punab et al., 2017); however, its poor solubility, instability, and rapid metabolism limit its clinical application (Zheng et al., 2022). A thermosensitive hydrogel scaffold was engineered to encapsulate atRA, preventing its degradation and enabling sustained high-dose delivery *in vivo*, thereby enhancing spermatogonial differentiation, completion of meiosis, and release of mature sperm—a novel tissue-engineering approach for male infertility (Zarei et al., 2025).

Nonalcoholic Fatty Liver Disease (NAFLD) contributes to infertility. Although high-dose Quercetin (Que; ≥10 mg/kg/day) exerts anti-inflammatory, anti-apoptotic, and antioxidant effects in NAFLD (Khaki et al., 2010), pro-oxidant activity at higher doses may impair fertility (Ranawat et al., 2013). To address this, a bigel hydrogel composed of cottonseed oil, hempseed oil, and acacia gum was formulated for controlled Que release, attenuating its oxidative toxicity. In NAFLD rat models, this Que-loaded bigel significantly reduced sperm abnormalities and DNA fragmentation rates, restored serum testosterone levels, and enhanced spermatogenesis (Mousavi et al., 2021).

SCI induces oxidative damage mediated by Reactive Oxygen Species (ROS), which elevates sperm DNA fragmentation and impairs fertility (Gosálvez et al., 2022). Erythropoietin (EPO) demonstrates anti-apoptotic, antioxidant, and anti-inflammatory effects via activation of antioxidant enzymes and inhibition of lipid peroxidation but suffers from inefficient *in vivo* delivery (Zhang et al., 2022). A chitosan/alginate hydrogel was developed for local EPO delivery following SCI. In treated rats, sperm motility was significantly improved and testicular malondialdehyde levels were decreased, indicating that the EPO-loaded hydrogel enhances therapeutic outcomes for SCI-related infertility (Rasekh et al., 2025).

## 6 Applications of hydrogels in intelligent models

For complex challenges in treating urogenital diseases, innovations in biomedical engineering have driven the development of intelligent *in vitro* models with diverse applications. Hydrogel technologies overcome limitations of traditional systems, enabling mechanistic studies, functional reconstruction, defect repair, and reproductive tissue engineering in urogenital disorders.

**FIGURE 9:**
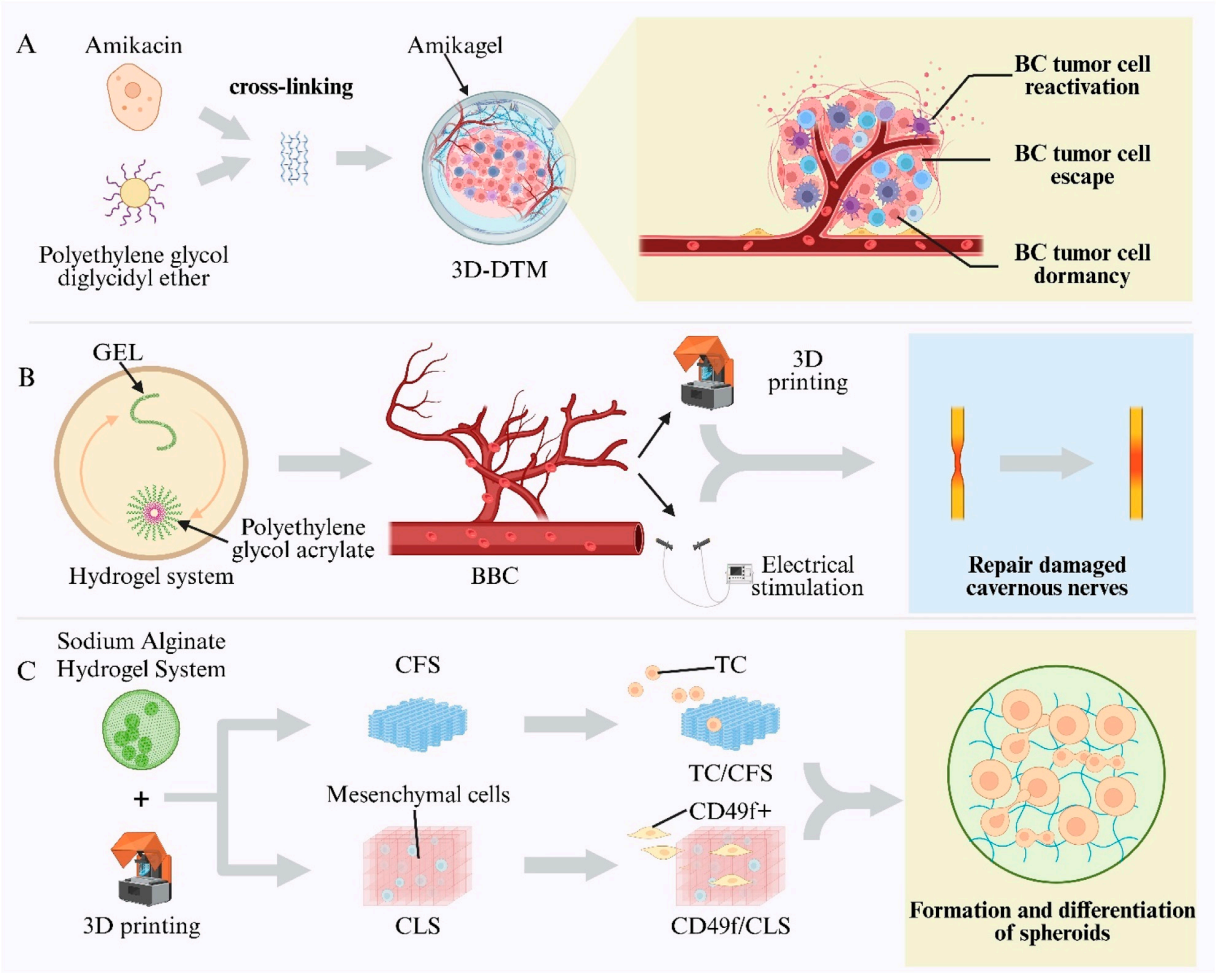
Hydrogel-enabled intelligent models in urogenital research. **(A)** Amikagel—amikacin cross-linked with Poly(Ethylene Glycol) Diglycidyl Ether (PEGDGE)—creates a Three-Dimensional Dormant-Tumour Micro-Environment (3D-DTM) for bladder-cancer studies. **(B)** Biomimetic Cavernosum Construct (BBC) printed from gelatin/Poly(Ethylene Glycol) Diacrylate (PEGDA) hydrogel replicates corporal biomechanics and aids nerve repair. **(C)** Sodium-alginate scaffolds printed as Cell-Free Scaffolds (CFS) or Cell-Laden Scaffolds (CLS) constructs support Testicular-Cell (TC) culture and spermatogonial differentiation.

A high postoperative recurrence rate is observed in BC, with non–muscle-invasive BC exhibiting recurrence rates of up to 90% after transurethral resection (Aldousari and Kassouf, 2010). Emerging evidence implicates chemotherapy-evasive dormant cancer cells in this process (Kottke et al., 2013; Páez et al., 2012). Conventional two-dimensional and spheroid cultures fail to replicate the dynamic dormancy microenvironment *in vivo*, including cell-cycle arrest, drug resistance, and reactivation phases (Aguirre-Ghiso, 2007). Moreover, standard chemotherapies target proliferating cells and are ineffective against G_0_/G_1_-arrested dormant cells (Wells et al., 2013). Thus, advanced *in vitro* systems are required to study dormancy mechanisms and to facilitate targeted therapy development (Knuchel et al., 1989). To address this need, a 3D Tumor Microenvironment Model (3D-DTM) was established using an amikacin-based hydrogel (“Amikagel”), in which amikacin was cross-linked with Polyethylene Glycol Diglycidyl Ether (PEGDGE) at controlled densities. This system faithfully mimicked BC cell dormancy, escape, and reactivation *in vitro*, providing a platform for anticancer drug screening, enhancing drug sensitivity, and eradicating dormant tumor cells (Figure 9A) (Pavan Grandhi et al., 2017).

Vascular ED is a leading cause of organic ED, often resulting from impaired cavernosal arterial inflow and inadequate venous occlusion (Montorsi et al., 2005; Shamloul and Ghanem, 2013). However, no physiologically relevant *in vitro* model of the corpora cavernosa has existed, hindering studies of cavernosal biomechanics, pathology, and repair. To overcome this gap, a BBC was fabricated via digital light processing 3D printing using Gelatin (GEL) and Polyethylene Glycol Diacrylate (PEGDA) hydrogels. The biomimetic cavernosal model (BBC) exhibited an elastic modulus and tensile strength comparable to native tissue, with >90% recovery after cyclic stretching. By simulating venous occlusion, the model validated key hemodynamic principles of erection and achieved *in vitro* rigidity. Furthermore, BBC scaffolds seeded with cavernous nerve-like constructs and subjected to electrical stimulation were implanted into rabbit and porcine cavernous defects, resulting in restored erectile function and enabling spontaneous mating (Figure 9B) (Wang Z. et al., 2025).

3D bioprinting has been applied to reproductive tissue engineering to replicate spermatogenesis *in vitro* (Martinovitch, 1937). Sodium alginate hydrogels serve as natural polymers for 3D bioprinting (Yokonishi et al., 2013; Jørgensen et al., 2014). Using an alginate-based hydrogel system, Cell-Free Scaffolds (CFS) and Cell-Laden Scaffolds (CLS) were fabricated (Aljohani et al., 2018). Unsorted prepubertal Testicular Cells (TCs) were seeded into the macropores of CFS to create single-cell compartments (TC/CFS), while CD49f^+^ epithelial cells, enriched by magnetic sorting, were seeded into CLS pores to form dual-cell compartments (CD49f^+^/CLS). Both scaffold types supported spheroid formation and germ-cell differentiation within their pores: 66% of TC/CFS constructs and 100% of CD49f^+^/CLS constructs contained postmeiotic germ cells, and elongated spermatids were detected in some constructs. These results validate the feasibility of 3D bioprinting for reproductive tissue engineering and provide a programmable platform for constructing biomimetic testicular microenvironments and studying cell–cell interactions (Figure 9C) (Baert et al., 2019).

## 7 Challenges and limitations

Hydrogels, owing to their distinctive properties and drug-loading capabilities, have demonstrated a broad spectrum of potential applications in the diagnosis and treatment of male urological diseases. Nonetheless, a review of current investigations reveals that their practical application encounters multiple challenges and limitations (Sánchez-Cid et al., 2022; Perez-Valle et al., 2020). These include an incongruity between the intrinsic properties of existing hydrogel systems and the complex therapeutic requirements of urological pathologies, restricted functionality of hydrogel-based systems within specific pathological microenvironments, unresolved issues related to the multi-targeted, synergistic control of drug delivery, as well as a paucity of long-term clinical data and standardized fabrication protocols.

Presently, the utilization of hydrogels in certain male reproductive disorders remains in the exploratory phase, with limited research particularly in the context of rare male malignancies such as penile and testicular cancers. In the realm of tissue engineering, current natural hydrogel materials exhibit significant deficiencies in maintaining morphological stability and structural integrity within the highly dynamic settings of urethral, penile, or bladder reconstruction. Conversely, synthetic hydrogels often face biocompatibility challenges (Hu et al., 2024). Although some studies have attempted to combine the advantageous features of both to engineer hydrogels with desirable elastic moduli suitable for tissue regeneration, such composite strategies are yet to be standardized, and their long-term stability remains inadequately validated. Moreover, current hydrogel-based tissue-engineering technologies still confront multi-faceted, complex hurdles on the path toward human clinical translation (Forouzandegan et al., 2025). The primary barrier is the considerable discrepancy between the human pathological microenvironment and the conditions recreated in existing experimental models. Animal models rarely reproduce the inflammation, fibrosis and latent injuries that may arise *in vivo*, making it difficult to guarantee ideal clinical outcomes (Kumari and Goyal, 2024). Compounding this problem is the severe paucity of data on inter-individual variability and long-term safety.

Furthermore, the bioresponsive properties of most hydrogel systems are heavily dependent on their microenvironment. For instance, in Erectile Dysfunction (ED) therapy, conductive hydrogels (e.g., GACM) are designed to preserve adhesive, conductive, and anti-inflammatory functions, which are notably influenced by cellular responses in the perilesional neural tissue. However, in male urological pathologies, the microenvironment tends to be highly complex, characterized by ischemia, hyperosmotic urine, or tumor heterogeneity. Whether such microenvironmental factors precipitate functional impairments of hydrogels warrants further investigation.

In the domain of drug delivery, current strategies predominantly rely on monolithic targeted approaches controlling the release of specific agents—such as thermosensitive release of Doxorubicin (DOX) or rapamycin, or zinc-triggered self-assembly for Docetaxel (DTX) delivery. However, in clinical practice, particularly in combined modalities involving immunotherapy, chemotherapy, and molecular targeting, there is a demand for multi-temporal, multi-site, and multi-pathway coordinated release systems. Currently, no hydrogel platform has been developed that precisely meets these multifaceted therapeutic requirements.

Regarding safety profiles, while existing research indicates that hydrogels possess high reliability, the majority of investigations in the context of male reproductive health remain confined to animal models, with limited progression into extensive clinical trials. At present, the only well-established clinical use centres on hydrogel spacers in prostate-cancer radiotherapy, and the scarcity of large-scale trials likely reflects the immaturity of hydrogel technology, technical limitations and low clinical acceptance. Moreover, scaling up hydrogel production encounters technical obstacles that hinder widespread clinical application and quality control. Because these systems require precise synthetic methods and advanced polymer engineering, the absence of standardised evaluation criteria and harmonised regulatory frameworks has slowed approval processes, introduced scalability issues, and—together with batch-to-batch variability and inconsistent fabrication performance—undermined industrial feasibility and reproducibility (Li S. et al., 2024). In addition, thermosensitive and supramolecular hydrogels demand strict temperature control during formulation and storage, leaving long-term preservation an unresolved obstacle (Klaewklod et al., 2015). Finally, Critical concerns pertaining to *in vivo* biocompatibility, especially long-term effects of degradation products on the male reproductive system, have yet to be thoroughly evaluated, and long-term follow-up data are lacking.

## 8 Conclusions and outlook

This review systematically summarizes research advances and application prospects of hydrogel technologies in andrology. As biomaterials characterized by high water content, excellent biocompatibility, and tunable physicochemical properties, hydrogels have demonstrated multidimensional utility in managing andrological disorders. In PC therapy, hydrogel spacers have been shown to physically separate the prostate and rectum, significantly reducing radiation-induced rectal toxicity during EBRT, while hydrogel-based drug-delivery systems enable precise locoregional release of chemotherapeutic agents. In BC, hydrogels combined with ICIs have established synergistic immunomodulatory regimens. For ED, hydrogel carriers have facilitated neurovascular repair and reconstruction. In male reproductive medicine, hydrogels have supported innovative sperm-selection platforms and *in vitro* spermatogenesis models. In urinary tract reconstruction, hydrogel scaffolds have been successfully applied to bladder and urethral repair, overcoming challenges posed by the urinary microenvironment. Moreover, hydrogels have enhanced diagnostic assays and optimized drug-delivery strategies across andrological diseases.

Future developments in hydrogel applications for andrology are expected to follow several key trends. First, smart, stimuli-responsive hydrogels will be engineered for multimodal responsiveness, integrating and reacting selectively to multiple physiological cues and disease-specific microenvironmental biomarkers. Second, integration with advanced manufacturing techniques—particularly three-dimensional (3-D) bioprinting—will enable customization of hydrogel implants to patient-specific anatomy and pathology. Moreover, the rapid advance of artificial intelligence is ushering in revolutionary breakthroughs in hydrogel research. Data-driven analytics, predictive modelling and optimisation algorithms are effectively tackling the core challenge of fine-tuning hydrogel properties and performance. In biomedical design, hydrogels must simultaneously balance multiple complex parameters—biocompatibility, mechanical robustness, degradation kinetics and drug-release behaviour. Traditional research relies on laborious trial-and-error experiments that are time-consuming and inefficient. AI, by swiftly processing vast datasets and accurately mapping structure–property relationships, enables reliable prediction of hydrogel behaviour and markedly accelerates the development of optimal systems. With AI’s continued empowerment, hydrogel technology is poised to transcend current constraints and drive a systemic revolution in precision medicine for andrology (Negut and Bita, 2023).

Third, hierarchical incorporation of bioactive molecules (e.g., growth factors, small-molecule mediators, non-coding RNAs) into hydrogel networks will facilitate spatiotemporally controlled release profiles. Finally, deep integration of hydrogels with stem-cell technologies is poised to drive transformative advances in andrological regenerative medicine, with future research focusing on designing hydrogel niches that direct stem-cell differentiation toward specialized male reproductive cell phenotypes.

Hydrogel technology in andrology is poised for clinical translation. Established hydrogel products—such as the SpaceOAR spacer—are being optimized for broader applications. As research advances, hydrogel platforms in male reproductive health are expected to transition from laboratory studies to clinical trials. In the long term, hydrogels are anticipated to serve as a primary modality for tissue engineering and regenerative medicine in andrology. Advancement of hydrogel technology will require interdisciplinary collaboration, with integrated innovation across materials science, biomedical engineering, andrology, and molecular biology. Rigorous application of translational medicine principles will facilitate the progression of hydrogel systems from bench to bedside, while partnerships with the medical-device and pharmaceutical industries will support industrialization and expand patient access.

In summary, owing to their distinctive physicochemical properties and superior biocompatibility, hydrogels offer expansive potential and growth prospects in andrology. Continued progress in materials science and fabrication methods is expected to enable hydrogels to provide novel solutions for the diagnosis, treatment, and prevention of andrological diseases, thereby improving patient quality of life and clinical outcomes.

## Glossary

ASCs: Adipose-Derived Stem Cells
ASC-Exo: Adipose-Derived Stem Cell Exosomes
atRA: All-Trans Retinoic Acid
BC: Bladder Cancer
BAM: Bladder Acellular Matrix
BTX-A: OnabotulinumtoxinA
CCA: Chitosan–Cyclodextrin Amphiphilic
CNP: Chronic Nonbacterial Prostatitis
CP: Chronic Prostatitis
CS: Chitosan
CHMA: Chitosan Methacryloyl
CMC: Carboxymethyl Cellulose
CFS: Cell-Free Scaffolds
DTM: Decellularized Testicular Matrix
DTX: Docetaxel
DOX: Doxorubicin
ECM: Extracellular Matrix
ED: Erectile Dysfunction
EBRT: External-Beam Radiotherapy
EMO: Emodin
EPO: Erythropoietin
GEL: Gelatin
GFMs: Gold Fiducial Markers
GCS: Glycol Chitosan
HA: Hyaluronic Acid
HC: Hemorrhagic Cystitis
ICIs: Immune Checkpoint Inhibitors
ICSI: Intracytoplasmic Sperm Injection
IGRT: Image-Guided Radiotherapy
IPN: Interpenetrating Polymer Networks
MRI: Magnetic Resonance Imaging
MRM: Male Reproductive Medicine
NAFLD: Nonalcoholic Fatty Liver Disease
NVB: Neurovascular Bundles
OAB: Overactive Bladder
Oxy: Oxybutynin Hydrochloride
PAA: Polyacrylic Acid
PC: Prostate Cancer
PEG: Polyethylene Glycol
PTV: Planning Target Volume
PVA: Polyvinyl Alcohol
PDGF-BB: Patelet-Derived Growth Factor-BB
PSA: Prostate-Specific Antigen
PUU: Polyurethane-Urea
RAP: Rapamycin
RME: Regenerative Microenvironment
ROS: Reactive Oxygen Species
SBRT: Stereotactic Body Radiotherapy
SFMA: Silk Fibroin Methacryloyl
SSCs: Spermatogonial Stem Cells
SQS: Spacer Quality Score
SHFe: Shikonin–Ferric Ion (Fe^3+^) Nanocomposite Hydrogel System
SHH: Sonic Hedgehog
SF: Silk Fibroin
T1D-ED: Erectile Dysfunction in Type 1 Diabetes Mellitus
UME: Urinary Microenvironment
VEGF: Vascular Endothelial Growth Factor
